# Identifying the structure-activity relationship of leelamine necessary for inhibiting intracellular cholesterol transport

**DOI:** 10.18632/oncotarget.16002

**Published:** 2017-03-08

**Authors:** Raghavendra Gowda, Gajanan S. Inamdar, Omer Kuzu, Saketh S. Dinavahi, Jacek Krzeminski, Madhu Babu Battu, Sreedhara R. Voleti, Shantu Amin, Gavin P. Robertson

**Affiliations:** ^1^ Department of Pharmacology, The Pennsylvania State University College of Medicine, Hershey, PA 17033, USA; ^2^ Department of Pathology, The Pennsylvania State University College of Medicine, Hershey, PA 17033, USA; ^3^ Department of Dermatology, The Pennsylvania State University College of Medicine, Hershey, PA 17033, USA; ^4^ Department of Surgery, The Pennsylvania State University College of Medicine, Hershey, PA 17033, USA; ^5^ The Penn State Melanoma and Skin Cancer Center, The Pennsylvania State University College of Medicine, Hershey, PA 17033, USA; ^6^ Penn State Melanoma Therapeutics Program, The Pennsylvania State University College of Medicine, Hershey, PA 17033, USA; ^7^ Foreman Foundation for Melanoma Research Laboratory, The Pennsylvania State University College of Medicine, Hershey, PA 17033, USA; ^8^ Drug Discovery Research Laboratory, INDRAS Private Limited, Hyderabad, India 500040

**Keywords:** melanoma, leelamine, abietic acid, structure-activity relationship, AKT

## Abstract

Leelamine is an anticancer chemotherapeutic agent inhibiting intracellular cholesterol transport. Cell death mediated by leelamine occurs due to the lysosomotropic property of the compound, its accumulation in the lysosome, and inhibition of cholesterol transport leading to lack of availability for key processes required for functioning of cancer cells. The present study dissects the structure-activity-relationship of leelamine using synthesized derivatives of leelamine and abietic acid, a structurally similar compound, to identify the moiety responsible for anti-cancer activity. Similar to leelamine, all active derivatives had an amino group or a similar moiety that confers a lysosomotropic property to the compound enabling its accumulation in the lysosome. Active derivatives inhibited intracellular cholesterol transport and hindered xenografted melanoma tumor development without obvious systemic toxicity. In silico studies suggested that active derivatives accumulating in lysosomes bound to NPC1, a protein responsible for cholesterol export from the lysosome, to inhibit its activity that then caused accumulation, and lack of cholesterol availability for other key cellular activities. Thus, active derivatives of leelamine or abietic acid maintained lysosomotropic properties, bound to NPC1, and disrupted cellular cholesterol transport as well as availability to retard tumor development.

## INTRODUCTION

Malignant melanoma is the most deadly form of skin cancer due to its high metastatic nature and propensity for developing resistance to chemotherapeutic agents [1]. V600E-BRAF inhibitors, Zelboraf and Tafinlar and a MEK inhibitor Mekinist have been approved by the FDA for treating patients with MAPK pathway activation [2]. However, these targeted therapeutic approaches are hindered by drug resistance that leads to development of more aggressive recurrent disease [1]. Studies showed compensatory reactivation of MAPK signaling through the activation or expression of various proteins in all parts of this pathway, which aids the development of drug resistance [3]. Therefore, new compounds are needed that inhibit multiple points in the same and different pathways to decrease the possibility of resistance development.

Multiple mechanisms promoting increased cholesterol levels in cancer cells have been identified that promote cancer development [4–7]. Cellular cholesterol levels are maintained by transport of bound forms of low-density lipoprotein (LDL) from the cell membrane to lysosomes through the endocytic pathway where it is hydrolyzed to free cholesterol by acid lipase, and then transported to the cytosol by Niemann Pick type C proteins (NPC) [4, 8]. Mutation disrupting the functioning of NPC1 or NPC2 proteins cause a lipid storage disorder called Niemann Pick disease Type C, characterized by cholesterol accumulation in the lysosomes [9]. Loss of function mutations in acid sphingomyelinase (ASM) also causes type A and B forms of Niemann Pick disease due to impairment of sphingosine efflux from lysosomes, and accumulation of sphingolipid as well as LDL-derived cholesterol in lysosomes [10, 11].

Leelamine is a compound capable of targeting multiple oncogenic signaling pathways due to its disruption of cholesterol transport, which makes this essential lipid unavailable for the cellular activities [12, 13]. Leelamine is a weakly basic amine with lysosomotropic properties promoting its accumulation inside acidic cellular organelles, such as lysosomes [14]. Accumulation of leelamine in late endosomal or lysosomal cell compartments causes blockade of cholesterol transport from the lysosomes to cytoplasm leading to deficiency of free cholesterol [14]. Free cholesterol is a key mediator for diverse cellular processes, including receptor meditated endocytosis and endosome trafficking [4, 6, 8, 15]. Deficiency in available cholesterol due to trapping in the lysosome leads to inhibition of autophagic flux and receptor-mediated endocytosis [16, 17]. Inhibition of receptor-mediated endocytosis in-turn shutdown receptor tyrosine kinase signaling and inhibits the activation of downstream PI3K/AKT, STAT3 and MAPK signaling cascades [4, 8, 14].

The present study identifies the moiety of leelamine conferring its cholesterol transport inhibitor activity by dissecting the structure-activity-relationship (SAR) of the compound. First, the carboxyl group of abietic acid (an inactive naturally occurring analog of leelamine) was modified to various groups including esters and amines. Second, the primary amine of leelamine was modified to various secondary and tertiary amines or amides with varying steric bulkiness. Results demonstrated that the replacement of the carboxyl group of abietic acid with an amine moiety provided anti-melanoma cell killing activity to the compound, while replacement of the amine group of leelamine with acetamide blocked its activity. This implies that the basicity of the amine is the key component modulating its lysosomal accumulation, which enables cancer cell death by disrupting intracellular cholesterol transport [18, 19].

Active derivatives of leelamine or abietic acid accumulated in lysosomes to induce cellular vacuolization and inhibited cholesterol transport, causing blockade of receptor mediated endocytosis and autophagic flux. Shutdown of these processes in turn disrupted receptor mediated tyrosine kinase signaling thereby decreasing the activity of PI3K/AKT, STAT3, and MAPK pathways. The optimized abietic acid derivative compound 4a containing an amino group, inhibited the growth of preexisting xenografted melanoma tumors following oral administration by an average of 51% without affecting animal body weights or blood markers of major organ function. Furthermore, active derivatives that accumulated in lysosomes were bound to NPC1, a protein responsible for cholesterol export from lysosome to disrupt its functioning. Thus, the amino group of leelamine appears to be responsible for its accumulation in lysosomes where it binds to NPC1 to prevent cholesterol release into the cell, thereby conferring its cholesterol transport inhibitory activity.

## RESULTS

Leelamine is a natural product found in the bark of pine trees [12]. It has lysosomotropic properties leading to its accumulation in lysosomes, which subsequently inhibits cholesterol transport from these organelles [14]. In contrast, abietic acid is a naturally occurring close structural analog of leelamine that does not affect cancer cells survival in culture or in animals [12]. Structurally, abietic acid is a tricyclic diterpene with a carboxylic group and a non-aromatic C ring. Since, abietic acid lacks the prominent amino group present on leelamine and does not inhibit cholesterol transport, it was hypothesized that the amino group present in leelamine but absent from abietic acid might confer its anticancer activity [12–14].

**Figure 1:**
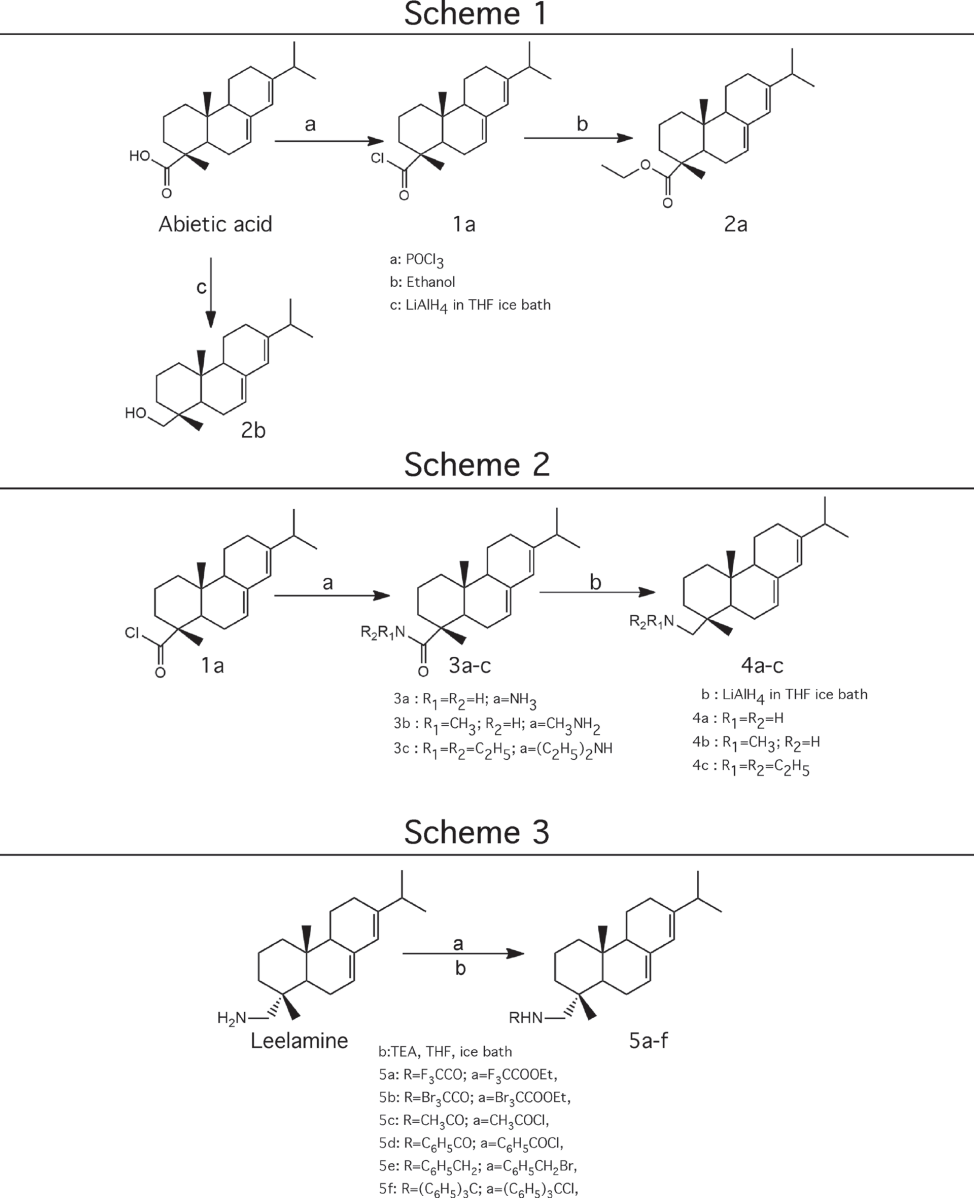
Schemes used for synthesizing abietic acid (Scheme 1 & 2) and leelamine (Scheme 3) derivative compounds

In order to dissect the chemical moiety in leelamine leading to its anticancer activity, derivative compounds of leelamine and abietic acid were synthesized. Analogs of abietic acid, 2a-b, 3a-c & 4a-c were synthesized by derivatizing the carboxylic acid group of abietic acid as represented in Figure 1, Schemes 1 and 2 and Table 1. Similarly, leelamine derivatives were synthesized by amidation of the amine group resulting in compounds 5a-f as represented in Figure 1, Scheme 3 and Table 2. Spectral and HPLC purity details for the key compounds of leelamine, 4a, 5a, 5c & 2a are provided in Supplementary Figure 1.

**Table 1:** Docking scores for active derivatives against NPC1 and NPC2

Names	Structures	Chemical properties				Melanoma cell killing efficacy (IC_50_ values)		
		pKa	LogD	LogD	PSA	FF2441	UACC 903	1205 Lu
			pH 7.4	pH 5.0		(Normal Cells)	(Melanoma cells)	
Abietic acid	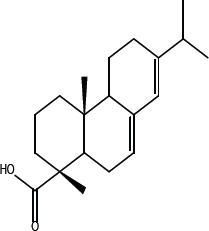	4.5	2.2	4.4	37.3	> 100	> 100	> 100
2a	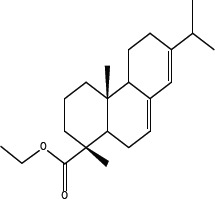	−7.1	5.4	5.4	26.3	> 100	> 100	> 100
2b	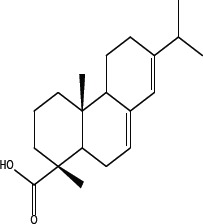	−1.2	44.0	4.4	20.2	> 100	52.6 ± 1.4	60.3 ± 1.6
3a	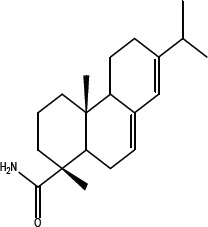	16.4	4.1	4.1	43.0	> 100	70.1 ± 1.8	81.0 ± 2.1
3b	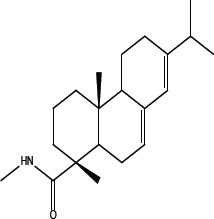	16.0	4.3	4.3	29.1	> 100	> 100	> 100
3c	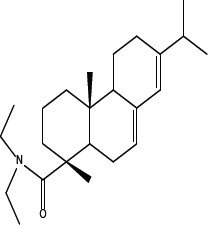	1.8	5.3	5.3	20.3	> 100	> 100	> 100
4a	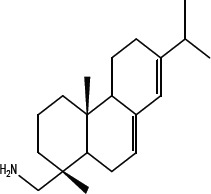	9.9	1.9	1.3	27.6	8.33 ± 0.4	2.13 ± 0.2	2.87 ± 0.1
4b	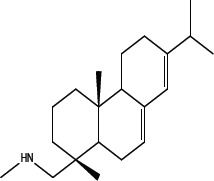	10.6	1.8	1.5	16.6	9.33 ± 1.2	2.36 ± 0.3	2.38 ± 0.2
4c	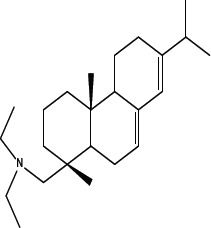	10.3	3.0	2.4	4.4	> 100	24.4 ± 1.4	24.3 ± 2.5

**Table 2:** Physicochemical and anti-melanoma activity of leelamine derivative compounds

Names	Structures	Chemical properties				Melanoma cell killing efficacy (IC_50_ values)		
		pKa	LogD	LogD	PSA	FF2441	UACC 903	1205 Lu
			pH 7.4	pH 5.0		(Normal Cells)	(Melanoma cells)	
Leelamine	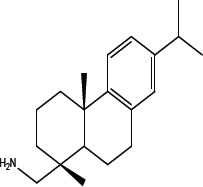	9.9	2.8	2.1	27.6	8.91 ± 0.6	1.35 ± 0.1	1.93 ± 0.2
5a	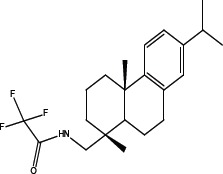	6.9	5.6	6.1	26.3	5.17 ± 0.1	1.28 ± 0.1	2.08 ± 0.1
5b	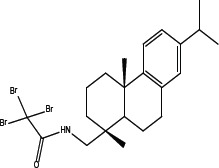	8.8	7.1	7.1	29.1	4.10 ± 0.2	1.01 ± 0.1	1.88 ± 0.2
5c	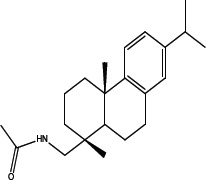	16.0	5.0	5.0	29.1	> 100	89.4 ± 3.3	> 100
5d	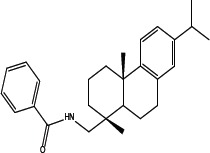	15.0	6.8	6.8	29.1	> 100	>100	> 100
5e	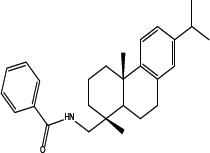	10.6	4.7	4.0	16.6	3.13 ± 0.4	6.05 ± 1.3	7.05 ± 0.7
5f	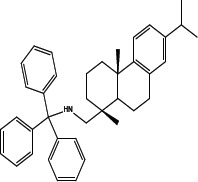	8.9	9.2	7.5	16.6	> 100	> 100	> 100

The carboxylic acid group of abietic acid was esterified to compound 2a (pKa −7.1; LogD 5.4) and reduced to alcohol containing compound 2b (pKa −1.2; LogD 4.4). Next, the carboxylic acid group of abietic acid was converted to various primary (compound 3a; pKa 16.4; LogD 4.3), secondary (compound 3b; pKa 16; LogD 4.3) and tertiary amides (compound 3c; pKa 1.83; LogD 5.3). Later, the amides were reduced to corresponding amines in presence of LiAlH_4_. Compound 3a was reduced to a primary amine 4a (pKa 9.9; LogD 1.9). Similarly, compound 3b (pKa 10.6; LogD 1.8) was reduced to secondary amine 4b and compound 3c was reduced to tertiary amine 4c (pKa 10.3; LogD 3.0).

Various amides of leelamine were synthesized in presence of triethanolamine. Compounds 5a, 5b & 5c were trifloro, tribromo substituted and unsubstituted acetyl amides of leelamine (pKa of 6.9, 8.8 and 16.0; LogD 5.6, 7.1 and 5.0 respectively). Compound 5d and 5e were benzoyl and benzyl amides of leelamine (pKa 15 and 10.6; LogD 6.8 and 4.7 respectively). Finally, compound 5f was a trityl substituted acetamide derivative of leelamine (pKa 8.9 and LogD 9.2).

### Identification of the moiety of leelamine conferring anti-melanoma cell killing activity by chemical modification of abietic acid

Abietic acid has no anticancer activity (IC_50_ = > 100 μmol/L) and at physiological pH, abietic acid would be highly charged (pKa 4.59), and not lysosome permeable (pH 4.8). Esterification of abietic acid (LogD at pH 7.4 is 2.2) to a more lipophilic compound 2a (LogD at pH 7.4 is 5.4) had no effect on its anticancer activity. Reduction of abietic acid to the alcohol 2b conferred relatively weaker anti-melanoma activity (IC_50_ of 52.6 and 60.3 μmol/L in UACC 903 and 1205 Lu cells, respectively). Conversion of abietic acid to an amide 3a conferred modest anti-melanoma activity (IC_50_ = 70.1 and 81 μmol/L in UACC 903 and 1205 Lu cells, respectively). Introducing mono or dialkyl substitution on the amide nitrogen produced 3b (methyl) and 3c (diethyl), completely abolished activity (IC_50_ = > 100 μmol/L in both UACC 903 and 1205 Lu cells). Amides did not improve activity due to neutrality and weak hydrogen bonding. Reduction of the amides 3a-c to basic amines 4a-c resulted in potent analogs 4a-b and modestly active 4c. The amines in 4a & 4b likely form hydrogen bonds at the active sites. 4a (IC_50_ of 2.1 and 2.9 μmol/L in UACC 903 and 1205 Lu cells, respectively) and 4b (IC_50_ of 2.3 and 2.3 μmol/L in UACC 903 and 1205 Lu cells, respectively) are the most potent abietic acid analogs. Whereas 4c (IC_50_ of 24.4 and 24.3 μmol/L in UACC 903 and 1205 Lu cells, respectively) was the most highly basic among the amines, it had no active hydrogen necessary for hydrogen bonding and therefore had weak interactions. Thus, compounds made from abietic acid that had an amino-like moiety similar to that occurring on leelamine had an anti-cancer effect implying this group was important for this activity.

### Chemical modification of leelamine to eliminate its cancer cell-killing efficacy in order to identify the chemical moiety required for activity

Derivatives of leelamine were synthesized by replacing the amino group with various chemical moieties and the effect on the anticancer activity of the resultant compounds were evaluated. The amino group of leelamine was replaced with a trifluoro acetyl group resulting in 5a (IC_50_ of 1.2 and 2.0 μmol/L in UACC 903 and 1205 Lu cells, respectively) and with a tribromo acetyl group resulting in 5b (IC_50_ of 1.0 and 1.8 μmol/L in UACC 903 and 1205 Lu cells, respectively). This strategy maintained the IC_50_s of the compounds similar to that of leelamine. Conversion of the amine of leelamine to an acetyl group resulted in 5c, which lost activity (IC_50_ of 89.4 and > 100 μmol/L in UACC 903 and 1205 Lu cells, respectively). The acetyl group does not cause the polarization that occurs with the trifluoro acetyl group and hence did not have interactions required for optimal activity. Using a similar approach, benzoylation of the amino group of leelamine resulted in 5d, which as expected was inactive (IC_50_ of > 100 μmol/L at UACC 903 and 1205 Lu cells, respectively). Reducing the benzamide to the corresponding benzylamine in compound 5e showed a modest decrease in the IC_50_ value (IC_50_ of 6.0 and 7.0 μmol/L in UACC 903 and 1205 Lu cells, respectively). One of the easiest ways in which the accessibility of the amino group can be blocked is by introduction of a bulkier moiety such as a triphenylmethyl or trityl group. When a trityl group was placed on the amine of the leelamine, the compound 5f ceased to be active (IC_50_ of > 100 μmol/L for UACC 903 and 1205 Lu cells, respectively), which likely occurs due to steric hindrance. Thus, compounds made from leelamine that eliminated the amino group or its charge tended to loose activity suggesting the importance of this moiety for its activity.

### Active leelamine and abietic acid derivatives retain lysosomotropic activity

**Figure 2:**
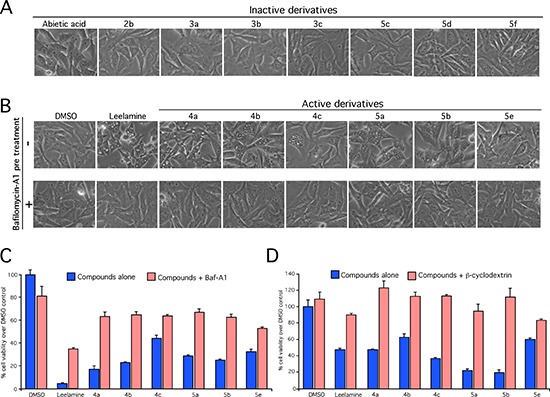
Lysosomotropism mediated by the amino group of leelamine is needed for vacuolization in cancer cell death (**A** and **B**) Lysosomotropism was measured by assessing the induction of cellular vacuolization by light microscopy of UACC 903 cells following treatment with active and inactive derivatives of abietic acid and leelamine alone or in combination with BafA1. (**C** and **D**) Viability of melanoma cells was assessed by MTS assay of cells treated with active derivatives 4a, 4b, 4c, 5a, 5b and 5e in the absence or presence of V-ATPase inhibitor BafA1 or β-cyclodextrin, which functions to remove cholesterol from the cells.

The vacuolar proton pumps (V-ATPase) establish the proton gradient in acidic organelles [18, 19]. This occurs in lysosomes, endosomes and secretory vesicles and drives the accumulation of lysosomotropic compounds in these organelles, which can be evidenced as cellular vacuoles [18, 19]. Inhibition of pumps can therefore be observed as prevention of cellular vacuolization following treatment with lysosomotropic compounds [19]. To assess whether active compounds retained lysosomotropism, leading to cholesterol accumulation in lysosomes as evidenced by vacuoles in cells, UACC 903 cells were treated with 10 nmol/L of Bafilomycin A1 (BafA1), a highly potent V-ATPase inhibitor, prior to the treatment with the compounds and cellular vacuolization examined [14]. BafA1 treatment prevented vacuolization of cells mediated by active leelamine and abietic acid derivatives, as occurs with control leelamine (Figure 2A and 2B). To confirm that lysosomotropic property of the active derivatives contributed to the activity, effect on cell viability was then investigated following co-treatment of BafA1 or β-cyclodextrin, which binds to and removes cholesterol from the cells. Co-treatment of 10 nmol/L BafA1 (Figure 2C) or 1 mM of β-cyclodextrin (Figure 2D) with active derivatives (4a-4c, 5a, 5b & 5e) reversed compound mediated cell death suggesting behavior similar to that exhibited by leelamine. In contrast, all inactive derivatives, failed to induce either vacuolization or death of UACC 903 cells even at high concentrations. Therefore, the amine group of leelamine appeared to confer the lysosomotropic activity to the compound, subsequently triggering cell death [14].

### Active leelamine and abietic acid derivatives inhibit cellular endocytosis and autophagic flux

**Figure 3:**
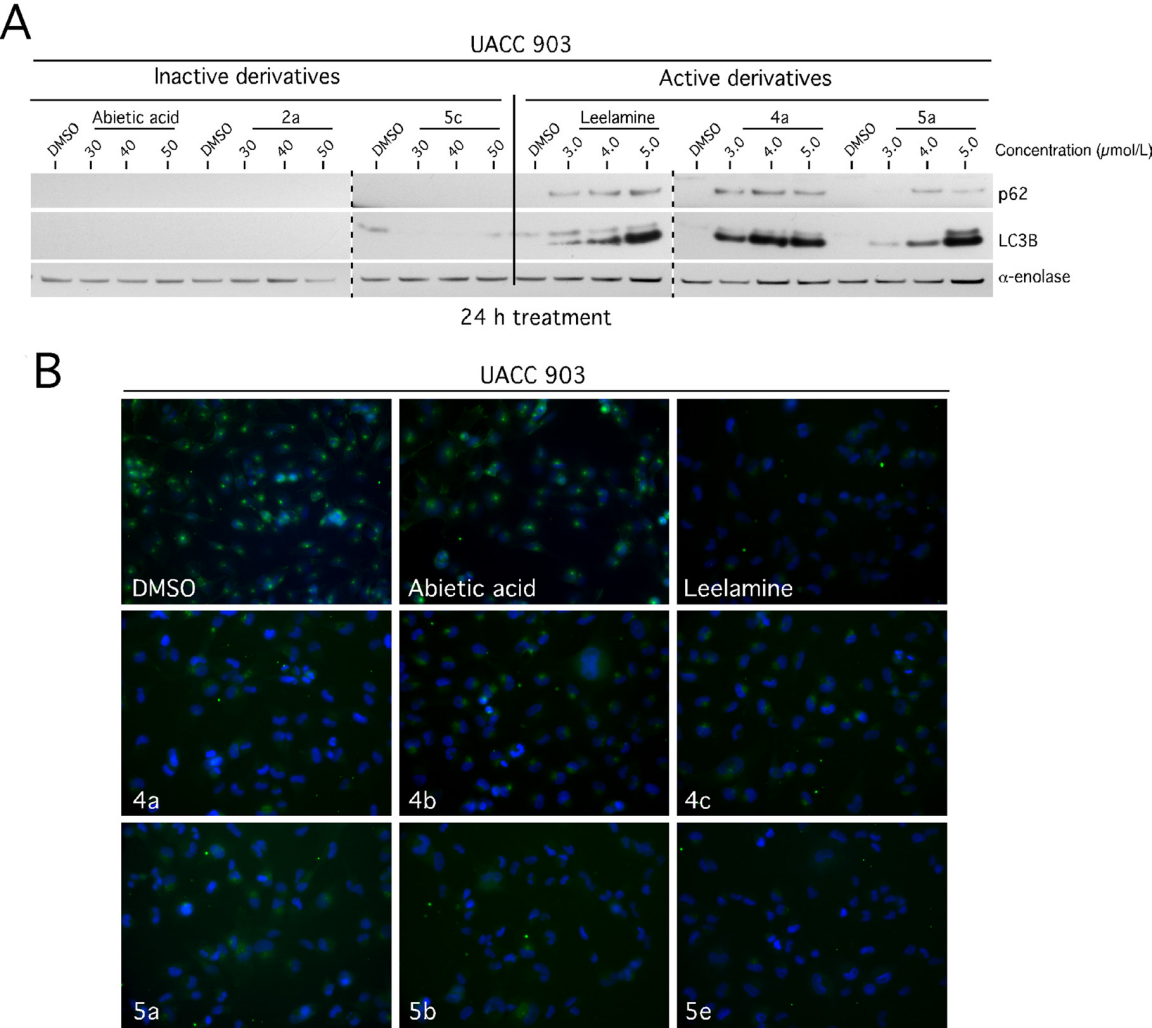
Leelamine and abietic acid derivatives containing an amino-group-like-moiety inhibited autophagic flux and cellular endocytosis (**A**) Active derivatives dose dependently induced accumulation of p62 and LC3B proteins indicating inhibition of autophagic flux in a manner similar to that of control leelamine. (**B**) Receptor mediated uptake of Alexa Fluor 488 conjugated transferrin protein, visualized by fluorescence miscopy, was inhibited with active derivatives in a manner similar to that occurring with leelamine. Abietic acid and DMSO vehicle served as a negative control.

The lysosomotropic property of leelamine disrupts cholesterol transport making this essential lipid unavailable to cells, which subsequently inhibits receptor mediated endocytosis and autophagic flux to shutdown the activity of key oncogenic pathways [12–14]. The effect of the derivative compounds on autophagic flux and related signaling pathways were examined by Western blotting of cells treated with inactive (Abietic acid, 2a, 5c) and active (Leelamine, 4a, 5a) derivatives. Similar to leelamine, active derivatives dose dependently induced accumulation of p62 and LC3B proteins indicating inhibition of autophagic flux (Figure 3A). Active derivatives of 4a-c, 5a, 5b & 5e blocked cellular endocytosis, which did not occur with inactive derivatives or control abietic acid, measured through Alexa flour conjugated transferrin protein uptake (Figure 3B).

### Active derivatives of leelamine and abietic acid bind to NPC1 to limit cholesterol interaction and transport

**Figure 4:**
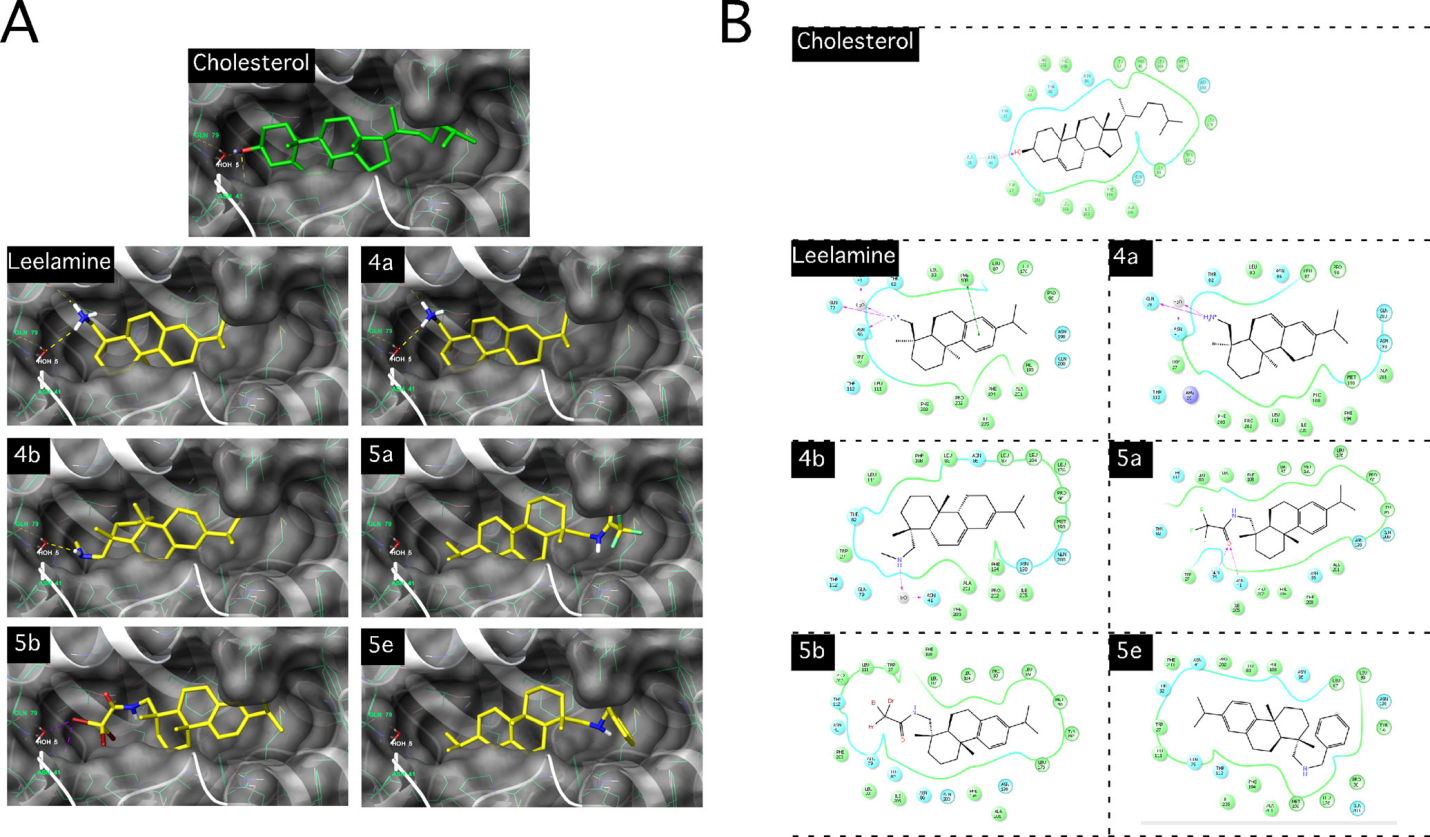
Active derivatives were capable of lysosomotropism and interacted with NPC1 in a manner similar to that of cholesterol and leelamine (**A**) Visualization of the predicted docking of cholesterol, leelamine and active derivatives in the cholesterol-binding pocket of NPC1 protein (PDB ID: 3GKI). (**B**) Two-dimensional interaction of cholesterol, leelamine and active derivatives in the cholesterol-binding pocket of NPC1.

NPC1 and NPC2 proteins interact to export free cholesterol from lysosomes [4, 15, 20]. Loss of function of either of the proteins results in lysosomal cholesterol accumulation appearing as cellular vacuolization [4, 15, 20]. To evaluate whether leelamine (accumulating in lysosomes) might interact with NPC1 and/or NPC2, molecular docking analysis of NPC1 and NPC2 proteins were performed. For NPC1, Asn41 and Gln79 form direct hydrogen bonds with the 3β-hydroxyl group of cholesterol, and a water-mediated indirect interaction with Glu30 (Figure 4A). Cholesterol docking simulations gave a dock score (DS) −14.35 and utilized low binding energy (BE) i.e −156.64 kCal/mol (Supplementary Table 1). However, NPC2 dock score with cholesterol was −11.01 with a binding energy of −140.33 kCal/mol, signifying a higher affinity of cholesterol with NPC1 than NPC2 (Supplementary Figure 2). Leelamine showed similar hydrophobic interactions as cholesterol with NPC1 and a H-bond was formed by the polar amine with the Gln79 backbone and water mediated H-bonds with Glu30 and Asn41 leading to a dock score of −11.21 and binding energy of −85.34 kCal/mol (Figure 4A). In contrast, leelamine did not have hydrogen bonding with NPC2 (Docking score of −9.25 with binding energy of −81.16 kCal/mol) (Supplementary Figure 2). Thus, leelamine seems to interact with NPC1 and to a significantly lesser extent with NPC2 to disrupt cholesterol transport.

Docking of active compounds with NPC1 was next examined to determine whether they acted in a similar manner to that of leelamine. Abietic acid or inactive derivatives were not considered for these studies since they would not accumulate in the lysosomes and therefore not have access to the NPC proteins. However, docking studies with inactive abietic acid in NPC1 had a dock score of −6.29 with a binding energy of −65.09 kCal/mol and with NPC2, a dock score of −2.35 with a binding energy of −80.76 kCal/mol (Supplementary Figure 3). These scores were lower than occurred with leelamine but docking might occur although poorly if the compound were taken into the lysosome. However, since this does not occur it would not be able to bind the NPC proteins. In contrast to abietic acid, compound 4a has a score comparable with leelamine for both NPC1 (DS: −10.65 and BE: −101.12 kCal/mol) (Figure 4A and Supplementary Table 1) and NPC2 (DS: −9.89 and BE: −95.87 kCal/mol) (Supplementary Figure 2). Presence of the polar amino group in both compounds, led to similar predicted binding interactions (hydrophobic and H-bond contacts) in both NPC1 and NPC2 active sites, as evidenced by similar anti-melanoma activity of 4a. Active derivatives (with IC_50_ ≤ 3.00 μmol/L) where the amino group of leelamine had been replaced with trifluoro acetamide (5a) and tribromo acetamide (5b) and an abietic acid derivative with a methanamine instead of acid group (4b) produced similar scores for both NPC1 and NPC2 (Figure 4A and Supplementary Table 1). Compounds 5a, 5b & 4b interacted with NPC1 polar residues with halogen or H-bonds, and had hydrophobic interactions with NPC1 similar to that observed for cholesterol and leelamine. Moderately active compound 5e has shown good NPC1 binding score with ample hydrophobic interactions but an absence of H-bonds (Figure 4A and Supplementary Table 1). 2D interaction pictures of all the compounds are depicted in Figure 4B for active derivatives. Thus, all active compounds had predicted interaction with NPC1 and NPC2 similar to that occurring for leelamine.

### Active derivatives of leelamine and abietic acid inhibited oncogenic pathway signaling

**Figure 5:**
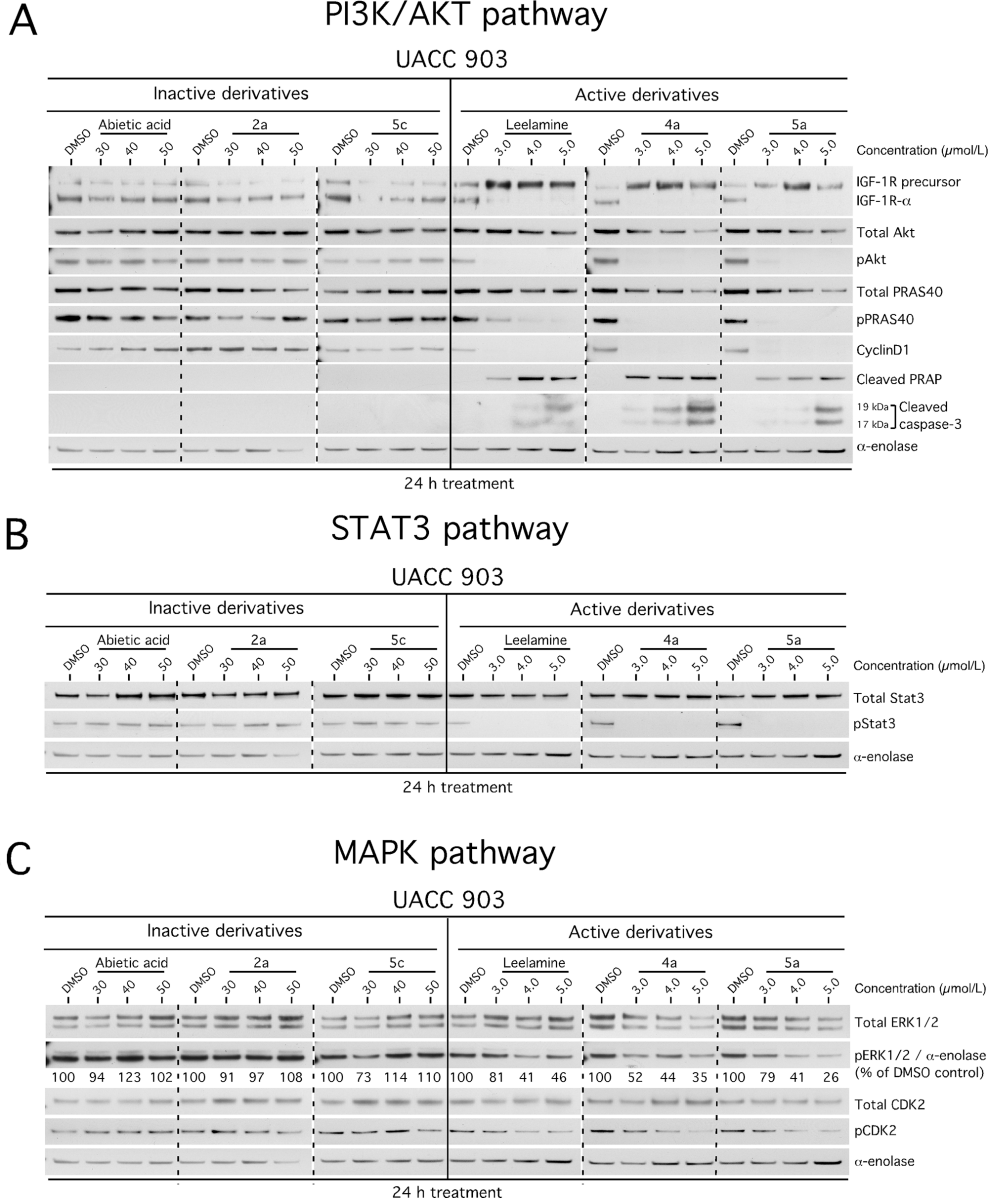
Active derivatives inhibit three key signaling pathways regulating melanoma development (**A**–**C**) Treatment with 3 to 5 μmol/L of active derivatives for 24 hours decreased PI3K/Akt (A), STAT3 (B) and MAPK (C) pathways in the UACC 903 melanoma cell line. 30–50 μmol/L treatments with of inactive derivatives did not affect the signaling cascades. Alpha-enolase served as a control for equal protein loading.

Decreased cholesterol levels in melanoma cells mediated by disruption of cholesterol transport by leelamine led to a subsequent shutdown of oncogenic signaling cascades promoting melanoma cell survival [12–14]. Representative active compounds 4a, 5a and inactive derivatives 2a, 5c were used to treat cells and effects on cell signaling pathways examined by Western blotting. Leelamine and active derivatives inhibited PI3K/AKT (Figure 5A), STAT3 (Figure 5B) and MAPK (Figure 5C) signaling pathways. In contrast, abietic acid and inactive derivatives did not affect these pathways even at concentrations as high as 50 μmol/L following 24 hours of treatment. Thus, active compounds tended to inhibit signaling cascades in a manner similar to that occurring with leelamine.

### An abietic acid derivative containing an amino group inhibited melanoma tumor development similar to that occurring with leelamine

The derivative compound 4a has similar Log D, pKa and PSA values compared to leelamine as well as similar efficacy for killing melanoma cells. An oral dosing of 80 mg/kg body weight was selected for 4a based on repeated dosing of leelamine (QOD) over 14 days. This dose and treatment schedule did not cause any weight loss but higher drug dosing regimens did (Supplementary Figure 4). In addition, the pharmacokinetics of leelamine has been undertaken in Swiss Webster mice. Leelamine was detectable in the serum of mice using LC MS/MS, following oral administration of 80 mg/kg body weight over a 36 hour period (Supplementary Figure 5). Therefore, the dose of compound 4a was selected as daily treatment with 80 mg/kg body weight in order to directly compare it to leelamine.

**Figure 6:**
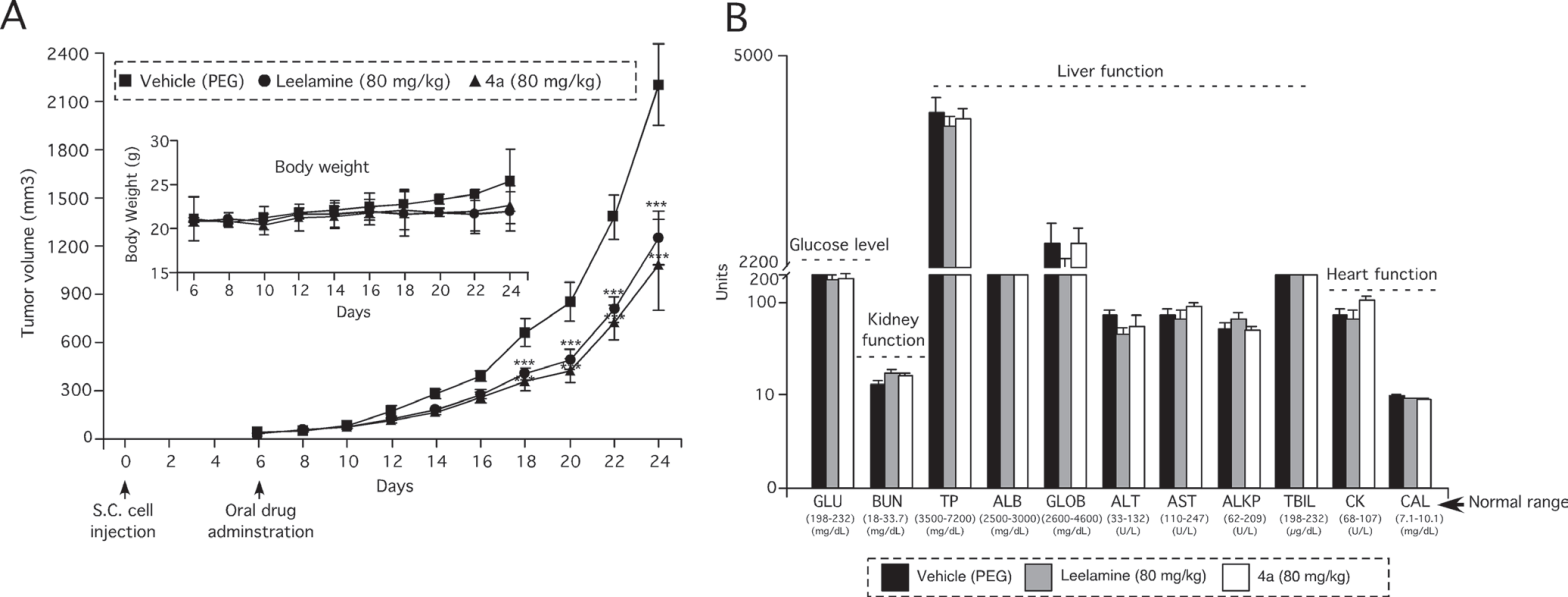
Amino group containing active derivative 4a of abietic acid inhibits melanoma tumor development with negligible toxicity (**A**) Active abietic acid derivative 4a inhibits melanoma tumor development by an average of 55%. Athymic nu/nu mice were s.c. injected with UACC 903 melanoma cells and six days later, treated orally daily with leelamine or compound 4a. 10% PEG was administered as vehicle control (4 mice/group; 2 tumors/mouse). Inset (A) No significant difference was observed in the body weight of mice following 24-days of treatment, indicating negligible toxicity. (**B**) Levels of blood biomarkers used to indicate major organ related toxicity, suggested negligible toxicity at the doses evaluated.

When a vascularized tumor had formed, mice were treated orally with 80 mg/kg of leelamine or compound 4a on a daily basis and tumor development was measured at 2-day intervals for 3 to 4 weeks (Figure 6A). Compound 4a reduced the tumor volume by 51% compared with the vehicle control, which was similar to leelamine mediated tumor inhibition. No significant differences between the body weights of mice were observed suggesting negligible toxicity. Furthermore, blood parameters (ALT, AST, ALKP, ALB, GLB, TPR, TBIL, BUN, GLU, CK and CAL) indicative of organ-related sub-chronic toxicity were measured at the end of study and fell in the normal range for this mouse species (Figure 6B).

## DISCUSSION

Leelamine is an intracellular cholesterol transport inhibitor with anticancer activity [14]. The lysosomotropic property of the compound leads to its accumulation in lysosomes where it then causes cholesterol accumulation, making this essential lipid unavailable for cancer cell functioning [14]. The SAR study detailed in this report identified the amino group of leelamine as being important for its lysosomotropic properties and anticancer efficacy. Conversion of the carboxylic acid in abietic acid to acid chloride, amide or esters did not increase the cancer inhibitory activity of the compound. Conversion to alcohol moderately increased activity, but conversion to amines led to activity equivalent to that of leelamine, which was observed for compounds 4a & 4b, indicating the importance of the amine group moiety for anticancer activity. However, tertiary amines, in compound 4c, were not active due to the absence of a H-bond donating capacity, which appears necessary for anticancer activity. Modifications of the primary amine in leelamine to amides reduced activity as seen in compounds 5c & 5d. However, there was no change in activity for trifluoro and tribromo substituted acetamides, demonstrating the polarizing capacity led to a favorable interaction. Furthermore, benzylation reduced the activity of leelamine and tritylation completely eliminated the activity, demonstrating that various degrees of steric hindrance at this site would alter the anti-cancer potential of the compound. Thus, the amine group on the polar side of the tricyclic ring moiety and sustained hydrophobic interactions mediated by the tricyclic ring moiety were essential for its anticancer activity. Modification of the primary amine reduced leelamine activity while conversion of the carboxylic acid group to amine in abietic acid increased efficacy.

ATP driven vacuolar type H^+^ pumps in cellular organelles such as lysosomes establishes the pH by pumping H^+^ ions into the lumen [21]. Lipophilic compounds such as leelamine easily diffuse through biological membranes in an unionized form but become trapped inside the interior acidic environment occurring in lysosomes due to decreased permeability upon protonation in the acidic environment [22, 23]. Leelamine and active derivatives of leelamine and abietic acid with pKa values between 6.9 to 10.6 exhibited lysosomotropism. Inhibitors of vacuolar H^+^-ATPase can suppress the activity of lysosomotropic compounds by inhibiting the acidification of cell compartments and preventing accumulation of these compounds [4]. Pre-treatment of melanoma cells with H^+^-ATPase inhibitor BafA1 suppressed cellular vacuolization and cell death mediated by active derivatives or the leelamine control, suggesting that lysosomotropism plays an essential role in the efficacy of compounds [14]. In addition, both BafA1 mediated inhibition of lysosomotropism and β-cyclodextrin mediated depletion of cholesterol suppressed cell death triggered by all active derivatives similar to the leelamine control, suggesting these compounds mediate cancer cell death through the same mechanism. This was subsequently confirmed by also showing that the active analogs inhibited cellular endocytosis, disrupted autophagic flux and shutdown oncogenic pathway signaling in cancer cells.

Lysosomal hydrolysis contributes to the mobilization of lipid droplet-associated cholesterol and NPC1 and NPC2 move cholesterol out of the lysosomes to make it available for cellular activities [24]. Mutation of NPC1 or NPC2 results in accumulation of cholesterol in the late endosomes or lysosomes leading to the autosomal recessive NPC disease [4, 15, 20]. Lysosomotropic compounds U18666A, clomiphene, imipramine, perphenazine and teraconazole cause cellular vacuolization similar to that observed with NPC disease [15, 20, 24–26]. NPC1 mutations limited the accumulation of lysosomotropic compounds in lysosomes, which could be restored by addition of GFP-tagged functional NPC1 protein [19]. Through molecular docking analysis, it seems that once leelamine or the active derivatives of leelamine or abietic acid accumulate in the lysosome, they interact with NPC1 to compete with its binding to cholesterol, thereby affecting export from the lysosome. For NPC1, Asn41 and Gln79 form direct hydrogen bonds with the 3β-hydroxyl group of cholesterol, and a water-mediated indirect interaction with Glu30. Leelamine similarly interacts with NPC1 through hydrophobic interactions and a H-bond was formed by the polar amine with the Gln79 backbone and water mediated H-bonds with Glu30 and Asn41. Active derivatives of leelamine or abietic acid similarly interacted with NPC1 having the same predicted hydrophobic and H-bond binding interactions. Thus, it is likely that once leelamine or active derivatives with similar lysosomotropic properties accumulate in lysosomes, there is potential for these compounds to interact with NPC1 to disrupt its interaction with and transport of cholesterol from the lysosome. Hence, it is possible that the direct target of leelamine is NPC1, leading to disruption of cholesterol transport in cancer cells.

Inhibition of cholesterol hemostasis and lack of availability in cancer cells following leelamine treatment halts receptor tyrosine kinase signaling and prevents the activation of downstream PI3K/AKT, STAT3 and MAPK signaling cascades [12–14]. These are key driver pathways constitutively activated in 50% to 70% of melanomas, functioning to reduce cellular apoptosis, increase proliferation and aid the invasive processes promoting melanoma progression. Similar to leelamine, the active derivatives inhibited the PI3K/AKT, STAT3 and MAPK signaling pathways. In contrast, abietic acid and inactive derivatives did not alter the activity of these pathways even at higher concentrations over prolonged periods of exposure.

In summary, this study demonstrates that the anticancer activity of leelamine was modulated by its primary amino moiety, which conferred lysosomotropic properties leading to its accumulation in the lysosome. Once in the lysosome, it then potentially interacted with NPC1 to disrupt cholesterol binding and its export from the lysosome mediated by NPC1. Lack of available cholesterol for the cancer cells then led to shutdown of receptor mediated endocytosis, endosome trafficking, and key oncogenic signaling cascades on which the cancer cells were dependent for survival.

## MATERIALS AND METHODS

### Cell line and culture conditions

Dr. Craig Myers, Penn State College of Medicine, Hershey, PA provided normal human fibroblast cell line FF2441. Mutant V600E-BRAF human melanoma cell line 1205 Lu was provided by Dr. Herlyn; Wistar Institute, Philadelphia, PA and UACC 903 was provided by Dr. Mark Nelson; University of Arizona, Tucson, AZ. Cell lines were maintained in a 37°C humidified 5% CO_2_ atmosphere incubator and periodically monitored for phenotypic, genotypic characteristics, and tumorigenic potential to validate and confirm cell line identity [12–14, 27–35].

### Synthesis and characterization of derivatives

Synthesized compound identity and purity were established using NMR (Nuclear Magnetic Resonance), MS (Mass Spectroscopy), TLC (Thin-layer Chromatography) and HPLC (High Performance Liquid Chromatography). NMR spectra were recorded using a Bruker Avance 500 MHz spectrometer. Chemical shifts (δ) were reported in parts per million downfield from the internal standard. The signals were quoted as s (singlet), d (doublet), t (triplet), m (multiplet), dd (doublet of doublet) and dt (doublet of triplet). Mass spectroscopic results were undertaken at the Core Facility of Penn State University. TLC was developed on aluminum-supported pre-coated silica gel plates (EMD, Germany). Column chromatography was conducted on neutral silica gel (60–200 mesh). Purity of the compound were established by HPLC using an HP-Agilent 1200 HPLC system on C18 column and all the compounds had purity of > 95% unless mentioned.

### Chemical synthesis of 1a: (1R, 4aR)-1, 4a-dimethyl-7-(propan-2-yl)-1, 2, 3, 4, 4a, 4b, 5, 6, 7, 10a-decahydrophenanthrene-1-carbonyl chloride

A mixture of abietic acid (1.0 eq. 9.95 mmoL), phosphorous (V) oxychloride (1.1 eq. 10.95 mmoL) and dimethyl formamide (DMF) (0.1 mL) was refluxed overnight in tetrahydrofuran (THF) (20 mL). The reaction mass was filtered under suction and concentrated under vacuum to give 2.98 g (93%) yellow oil. The compound was used immediately for the next step without any purification.

### Chemical synthesis of 2a: [(1R, 4aR)-1,4a-dimethyl-7-(propan-2-yl)-1, 2, 3, 4, 4a, 4b, 5, 6, 7,10a-decahydrophenanthren-1-yl] methanol

To a crude acid chloride 1a (1.0 eq. 3.11 mmoL), excess of ethanol was added and reaction was stirred for 2–4 hours. The reaction was washed with saturated sodium bicarbonate solution, followed by water and extracted with ethyl acetate. The organic layer was separated, dried over anhydrous sodium sulphate and evaporated under reduced pressure to get crude product. The crude product was purified over neutral silica and eluted with 10–20% ethyl acetate in hexanes to get 0.49 g (48%) of pure ester, 2a. HPLC purity- 85%; ^1^H NMR (CDCl_3_, δ ppm) 5.78 (s, 1H), 5.37–5.38 (d, 1H), 4.04–4.75 (m, 2H), 2.81–2.93 (m, 1H), 2.20–2.33 (m, 1H), 2.04–2.12 (m, 2H), 1.70–1.85 (m, 4H), 1.45–1.65 (m, 6H), 1.20–1.30 (m, 12H), 0.97–1.03 (m, 3H).

### Chemical synthesis of 2b: [(1R, 4aR)-1,4a-dimethyl-7-(propan-2-yl)-1, 2, 3, 4, 4a, 4b, 5, 6, 7,10a-decahydrophenanthren-1-yl] methanol

To a solution of abietic acid (1.0 eq. 3.30 mmoL) in THF (10 mL) at 0°C, LiAlH_4_ (4.15 eq. 13.69 mmoL) was added. The reaction was stirred overnight, treated with ethyl acetate at 0°C, followed by saturated ammonium chloride solution. The reaction was extracted with ethyl acetate, separated, dried over anhydrous sodium sulphate and evaporated under reduced pressure to get crude alcohol. The crude product was purified over neutral silica and eluted with hexanes to get 0.55 g (58%) of 2b. HPLC purity- > 95%; Mass = 286; ^1^H NMR (CDCl_3_, δ ppm) 5.78 (s, 1H), 5.40–5.41 (t, 1H), 3.46–3.49 (m, 1H), 3.14–3.24 (m, 1H), 2.19–2.24 (m, 1H), 2.01–2.08 (m, 2H), 1.80–1.88 (m, 2H), 1.51–1.60 (m, 6H), 1.37–1.41 (m, 2H), 1.21–1.26 (m, 2H), 1.03–1.05 (m, 1H), 1.00–1.02 (m, 6H), 0.89 (s, 3H), 0.83 (s, 3H).

### Chemical synthesis of 3a: (1R, 4aR)-7-isopropyl-1,4a-dimethyl-1,2,3,4,4a,4b,5,6,10,10a-decahydrophenanthrene-1-carboxamide

Excess ammonia gas was passed into a stirred mixture of dihydroabietic acid chloride 1a (1.0 eq. 9.28 mmoL) in dichloromethane at 0°C for 2–3 hours. The reaction was treated with 3N hydrochloric acid, followed by saturated sodium bicarbonate solution and finally washed with water. Organics were dried over anhydrous sodium sulphate and evaporated under vacuum to get a pale yellow product. The crude product was purified over neutral silica and elution with 20% ethyl acetate in hexanes to 35% ethyl acetate in hexanes to get 1.35 g (48%) of pure amide. HPLC purity- > 95%; Mass = 301.67, ^1^H NMR (CDCl_3_, δ ppm) 5.78 (s, 1H), 5.37–5.38 (t, 1H), 2.20–2.28 (m, 1H), 2.10–2.11 (m, 3H), 2.02 (s, 1H), 1.63–1.82 (m, 3H), 1.59–1.63 (m, 3H), 1.28–1.31 (m, 5H), 1.23–1.25 (m, 4H), 1.01–1.04 (m, 6H), 0.86 (s, 3H).

### Chemical synthesis of 3b: (1R, 4aR)-N, 1, 4a-trimethyl-7-(propan-2-yl)-1, 2, 3, 4, 4a, 4b, 5, 6, 10, 10a-decahydrophenanthrene-1-carboxamide

To a mixture of dihydroabietic acid chloride 1a (1.0 eq. 6.23 mmoL) in dichloromethane (30 mL), methylamine solution 2M in THF (1.1 eq. 6.85 mmoL) at 0°C was added and the reaction was stirred at room temperature overnight. The reaction mass was treated with diluted HCl, extracted with ethyl acetate, washed with brine, dried over anhydrous sodium sulphate and concentrated under vacuum to get colorless oil. The crude extract was purified over neutral silica and eluted with 20% ethyl acetate in hexanes to 35% ethyl acetate in hexanes to get 1.15 g (58%) of pure amide. HPLC purity- > 95% MS = 327.60 ^1^H NMR (CDCl_3_, δ ppm) 5.78 (s, 1H), 5.37–5.38 (t, 1H), 2.20–2.28 (m, 1H), 2.26 (s, 3H), 2.10–2.11 (m, 3H), 2.02 (s, 1H), 1.63–1.82 (m, 3H), 1.59–1.63 (m, 3H), 1.28–1.31 (m, 5H), 1.23–1.25 (m, 3H), 1.01–1.04 (m, 6H), 0.86 (s, 3H).

### Chemical synthesis of 3c: (1R, 4aR)-N, N-diethyl-1, 4a-dimethyl-7-(propan-2-yl)-1, 2, 3, 4, 4a, 4b, 5, 6, 10, 10a-decahydrophenanthrene-1-carboxamide

To a mixture of dihydroabietic acid chloride 1a (1.0 eq., 6.23 mmoL) in dichloromethane (30 mL), diethyl amine (1.1 eq. 6.85 mmoL) at 0°_C_ was added and the reaction was stirred at room temperature overnight. The reaction mass was treated with diluted HCl, extracted with ethyl acetate, washed with brine, dried over anhydrous sodium sulphate and concentrated under vacuum to get colorless oil. The crude extract was purified over neutral silica and elution with 20% ethyl acetate in hexanes to 35% ethyl acetate in hexanes to get 1.0 g (50%) of pure amide. HPLC purity- > 95%; MS = 357.90, ^1^H NMR (CDCl_3_, δ ppm) 5.77 (s, 1H), 5.38–5.39 (t, 1H), 3.47–3.51 (m, 2H), 3.33–3.72 (m, 2H), 2.17–2.23 (m, 3H), 2.06–2.08 (m, 3H), 1.80–1.90 (m, 2H), 1.61–1.63 (m, 2H), 1.32 (s, 3H), 1.21–1.26 (m, 6H), 1.12–1.14 (m, 6H), 1.00–1.03 (m, 6H), 0.85–0.98 (s, 5H).

### Chemical synthesis of dihydroabietylamines 4a-c

To a mixture of corresponding amides (1.0 eq. 0.33 mmoL) in dry THF (20 mL), 2M LiAlH_4_ in THF (4.0 eq. 1.33 mmoL) was added at 0–10°_C_ over the period of 5 minutes. The reaction was stirred for 4 hours (gradually allowing temperature to rise to room temperature). The reaction was treated with ethyl acetate, followed by saturated ammonium chloride solution, which was acidified with 3N hydrochloric acid. The reaction was then extracted with 3×10 mL ethyl acetate, washed with water and then with brine. The organics were dried over anhydrous sodium sulphate and removed under vacuum to get crude compound. The crude compound was treated with 2 M HCl in ether at 0°_C_ and then overnight at room temperature, solvent removed under vacuum and triturated with dry ether to get the corresponding hydrochlorides salts of the amine.

### Chemical synthesis of 4a: [(1R, 4aR)-1,4a-dimethyl-7-(propan-2-yl)-1,2,3,4,4a, 4b, 5,6,10,10a-decahydrophenanthren-1-yl] methanamine

Yield = 30%, HPLC purity- > 95%; MS = 288.2, ^1^H NMR (CDCl_3_, δ ppm) 5.78 (s, 1H), 5.37–5.38 (t, 1H), 2.67–2.69 (m, 1H), 2.42–2.44 (m, 1H), 2.20–2.28 (m, 1H), 2.10–2.11 (m, 3H), 2.02 (s, 1H), 1.63–1.82 (m, 3H), 1.59–1.63 (m, 3H), 1.28–1.31 (m, 5H), 1.23–1.25 (m, 4H), 1.01–1.04 (m, 6H), 0.86 (s, 3H).

### Chemical synthesis of 4b: {[(1R, 4aR)-1,4a-dimethyl-7-(propan-2-yl)-1,2,3,4,4a, 4b, 5,6,10,10a-decahydrophenanthren-1-yl] methyl}(methyl) amine

Yield = 38%, HPLC purity- > 95%; MS = 303, ^1^H NMR (CDCl_3_, δ ppm) 5.78 (s, 1H), 5.37–5.38 (t, 1H), 3.26 (s, 3H), 2.58–2.60 (m, 1H), 2.33–2.35 (m, 1H), 2.20–2.23 (m, 1H), 2.26 (s, 3H), 2.03–2.11 (m, 3H), 2.02 (s, 1H), 1.63–1.82 (m, 3H), 1.59–1.63 (m, 3H), 1.28–1.31 (m, 5H), 1.23–1.25 (m, 3H), 1.01–1.04 (m, 6H), 0.86 (s, 3H).

### Chemical synthesis of 4c: N-ethyl-N-(((1R, 4aR)-7-isopropyl-1, 4a-dimethyl-1,2,3,4,4a, 4b,5,6,10,10a -decahydrophenanthren- 1-yl)methyl) ethanamine

Yield = 35%, HPLC purity- > 95%; MS = 345, ^1^H NMR (CDCl_3_, δ ppm) 5.77 (s, 1H), 5.38–5.39 (t, 1H), 3.47–3.51 (m, 2H), 3.33–3.45 (m, 2H), 2.39–2.40 (m, 1H), 2.12–2.15 (m, 1H), 2.17–2.23 (m, 3H), 2.06–2.12 (m, 3H), 1.80–1.90 (m, 2H), 1.61–1.63 (m, 2H), 1.32 (s, 3H), 1.21–1.26 (m, 6H), 1.00–1.03 (m, 6H), 0.85–0.98 (s, 5H).

### Chemical synthesis of 5a: N- {[(1R, 4aS)-1, 4a-dimethyl-7-(propan-2-yl)-1, 2, 3, 4, 4a, 9, 10, 10a-octahydrophenanthren-1-yl] methyl}-2, 2, 2-trifluoroacetamide

To a solution of leelamine (1.0 eq. 6.23 mmoL) in THF (30 mL), triethylamine (TEA) (2.0 eq. 12.46 mmoL) was added at 0°_C_ followed by ethyl trifluoroacetate (1.1 eq. 6.85 mmoL) and the reaction was refluxed for 3–4 hours. The reaction mass was treated with diluted HCl, washed with water, dried over anhydrous sodium sulphate and concentrated under vacuum to get a colorless to pale yellow oil. The crude extract was purified over neutral silica and eluted with 10% ethyl acetate in hexanes to 20% ethyl acetate in hexanes to get 1.37 g (58%) of pure amide. MS = 396.42, 358.40, 381.47; ^1^H NMR (CDCl_3_, δ ppm) 7.19–7.21 (d, 1H), 7.03–7.05 (dd, 1H), 6.934–6.935 (d, 1H), 6.28 (bs, 1H), 3.30–3.32 (m, 2H), 2.94–2.99 (m, 1H), 2.82–2.89 (m, 2H), 2.33–2.36 (m, 1H), 1.74–1.88 (m, 4H), 1.42–1.53 (m, 3H), 1.29–1.30 (m, 1H), 1.25–1.28 (m, 6H), 1.01 (s, 3H), 0.87–0.90 (s, 3H).

### Chemical synthesis of 5b: N- {[(1R, 4aS)-1, 4a-dimethyl-7-(propan-2-yl)-1, 2, 3, 4, 4a, 9, 10, 10a-octahydrophenanthren-1-yl] methyl}-2, 2, 2-tribromoacetamide

To a solution of leelamine (1.0 eq. 6.23 mmoL) in THF (30 mL), TEA (2.0 eq. 12.46 mmoL) was added at 0°_C_ followed by ethyl tribromoacetate (1.1 eq. 6.85 mmoL) and the reaction was refluxed for 3–4 hours. The reaction mass was treated with diluted HCl, washed with water, dried over anhydrous sodium sulphate and concentrated under vacuum to get a colorless to pale yellow oil. The crude extract was purified over neutral silica and eluted with 10% ethyl acetate in hexanes to 20% ethyl acetate in hexanes to get 1.37 g (58%) of pure amide. MS = 396.42, 358.40, 381.47; 1H NMR (CDCl_3_, δ ppm) 7.19–7.21 (d, ^1^H), 7.03–7.05 (dd, 1H), 6.934–6.935 (d, 1H), 6.28 (bs, 1H), 3.30–3.32 (m, 2H), 2.94–2.99 (m, 1H), 2.82–2.89 (m, 2H), 2.33–2.36 (m, 1H), 1.74–1.88 (m, 4H), 1.42–1.53 (m, 3H), 1.29–1.30 (m, 1H), 1.25–1.28 (m, 6H), 1.01 (s, 3H), 0.87–0.90 (s, 3H).

### Chemical synthesis of 5c: N- {[(1R, 4aS)-1, 4a-dimethyl-7-(propan-2-yl)-1,2,3,4,4a, 9, 10, 10a-octahydrophenanthren-1-yl] methyl} acetamide

To a solution of leelamine (1.0 eq. 6.23 mmoL) in THF (30 mL) was added TEA (2.0 eq. 12.46 mmoL) at 0°_C_ followed by acetyl chloride (1.1 eq. 6.85 mmoL) and the reaction was stirred for 3–4 hours at room temperature. The reaction mass was treated with diluted HCl, washed with water, dried over anhydrous sodium sulphate and concentrated under vacuum to get a yellow oil, which solidified gradually. The crude extract was purified over neutral silica and subjected to elution with 10% ethyl acetate in hexanes to 20% ethyl acetate in hexanes to get 2.07 g (60%) of pure amide. HPLC purity- > 95% MS = 327.60, 369.40 corresponding to potassium adduct and 655.61 corresponding to dimer; ^1^H NMR (CDCl3, δ ppm) 7.17–7.18 (d, 1H), 6.99–7.01 (dd, 1H), 6.90 (s, 1H), 5.51 (bs, 1H), 3.22–3.25 (m, 1H), 3.08–3.12 (m, 1H), 2.90–2.95 (m, 1H), 2.79–2.86 (m, 2H), 2.28–2.31 (dt, 1H), 1.98 (s, 3H), 1.65–1.91 (m, 5H), 1.36–1.43 (m, 3H), 1.26 (s, 3H), 1.22–1.25 (m, 6H), 0.94 (s, 3H).

### Chemical synthesis of 5d: N-(((1R, 4aS)-7-isopropyl-1, 4a-dimethyl-1, 2, 3, 4, 4a, 9, 10, 10a-octahydrophenanthren-1-yl) methyl) benzamide

To a solution of leelamine (1.0 eq. 6.23 mmoL) in THF (30 mL) was added TEA (2.0 eq. 12.46 mmoL) at 0°_C_ followed by benzoyl chloride (1.1 eq. 6.85 mmoL) and the reaction was stirred for 3–4 hours at room temperature. The reaction mass was treated with diluted HCl, washed with water, dried over anhydrous sodium sulphate and concentrated under vacuum to get a yellow oil, which solidified gradually. The crude extract was purified over neutral silica and subjected to elution with 10% ethyl acetate in hexanes to 20% ethyl acetate in hexanes to get 2.07 g (60%) of pure amide. HPLC purity- > 95% MS = 390.51; ^1^H NMR (CDCl_3_, δ ppm) 7.72–7.73 (d, 2H), 7.47–7.50 (t, 1H), 7.40–7.43 (t, 2H), 7.18–7.23 (d, 1H), 6.98–6.99 (dd, 1H), 6.89 (s, 1H), 6.22 (s, 1H), 2.29–2.32 (d, 1H), 1.96–2.01 (m, 1H), 1.68–1.82 (m, 4H), 1.49–1.56 (m, 4H), 1.33–1.49 (m, 2H), 1.21–1.26 (m, 10H), 1.02 (s, 3H).

### Chemical synthesis of 5e: N-benzyl-1-((1R, 4aS)-7-isopropyl-1, 4a-dimethyl-1, 2, 3, 4, 4a, 9, 10, 10a-octahydrophenanthren-1-yl) methanamine

To a solution of leelamine (1.0 eq. 6.23 mmoL) in THF (30 mL) was added TEA (2.0 eq. 12.46 mmoL) at 0_°_C followed by benzyl bromide (1.1 eq. 6.85 mmoL) and the reaction was refluxed for 3–4 hours. The reaction mass was washed with water, dried over anhydrous sodium sulphate and concentrated under vacuum to get colorless to pale yellow oil, which when triturated with dry ether gave white precipitate. Ether was decanted; precipitate was washed with ether several times to get 55 mg (2%) of pure amine. HPLC purity- > 95% MS = 376.73 (M+1); ^1^H NMR (CDCl_3_, δ ppm) 7.54–7.55 (d, 2H), 7.32–7.34 (d, 3H), 6.97–6.99 (d, 1H), 6.78–6.85 (dd, 1H), 6.71 (s, 1H), 4.07 (s, 2H), 2.73–2.78 (m, 1H), 2.63–2.67 (m, 2H), 2.43–2.46 (d, 1H), 1.67–1.70 (m, 1H), 1.58–1.61 (m, 3H), 1.48 (s, 3H), 1.30–1.36 (m, 3H), 1.17 (m, 4H), 1.06–1.10 (m, 6H), 0.97 (s, 3H).

### Chemical synthesis of 5f: {[(1R, 4aS)-1, 4a-dimetyl-7-(propan-2-yl)-1, 2, 3, 4, 4a, 9, 10, 10a-octahydrophenanthren-1-yl] methyl} (triphenylmethyl) amine

To a solution of leelamine (1.0 eq. 6.23 mmoL) in THF (30 mL) was added TEA (2.0 eq. 12.46 mmoL) at 0°_C_ followed by triphenylmethyl chloride (1.1 eq. 6.85 mmoL) and the reaction was stirred for 3–4 hours at room temperature. The reaction mass was washed with water, dried over anhydrous sodium sulphate and concentrated under vacuum to get colorless to pale yellow oil. The crude was purified over neutral silica and elution with 10% ethyl acetate in hexanes to 20% ethyl acetate in hexanes to get 2.1 g (63%) of pure amine. HPLC purity- > 95% MS = 286.50 (M+1) (minus the trityl group); ^1^H NMR (CDCl_3_, δ ppm) 7.54–7.55 (d, 2H), 7.32–7.34 (d, 3H), 6.97–6.99 (d, 1H), 6.78–6.85 (dd, 1H), 6.71 (s, 1H), 4.07 (s, 2H), 2.73–2.78 (m, 1H), 2.63–2.67 (m, 2H), 2.43–2.46 (d, 1H), 1.67–1.70 (m, 1H), 1.58–1.61 (m, 3H), 1.48 (s, 3H), 1.30–1.36 (m, 3H), 1.17 (m, 4H), 1.06–1.10 (m, 6H), 0.97 (s, 3H).

### Protein homology modeling

Leelamine and abietic acid derivatives were drawn in the ChemDraw application and 2D sketcher tool in Maestro 10.1. Molecular docking studies for all these molecules were performed using GLIDE (Grid Ligand Docking with Energetics) 6.6 in Maestro 10.1 [36]. Protein structure homology model of hNPC2 was humanized from the bovine NPC2 crystal structure using the multiple sequence viewer tool in maestro, by utilizing mutate residue option followed by minimizations, and protein structure validation was completed by Prime 3.9 protein modeling tool in Maestro 10.1 [37]. Due to unavailability of original crystal structure for the Human NPC2 protein, a model structure using homology modeling was developed [38]. Crystal structure of the bovine NPC2 (PDB: 2HKA) was humanized by substituting the amino acid residues to attain the exact sequence match for the human NPC2 amino acid sequence. Sequence alignment information was achieved from CLUSTALO (1.2.1) and BLASTp online tools [39]. Protein minimizations and validations were performed using the prime 3.9 application from Maestro 10.1 software after conversion of bovine NPC2 to human NPC2 structure [37]. The alignment of amino acids in bovine and human NPC2 is shown in Supplementary Figure 6.

### Binding interactions analysis

Binding interactions of leelamine and abietic acid derivatives with NPC1 (PDB: 3GKI) and NPC2 (PDB: 2HKA) proteins were analyzed using the GLIDE docking application in Maestro 10.1 software [36]. Grids were generated based on the location of the crystal ligand-binding site (cholesterol site in NPC1 and cholesterol sulfate site in NPC2), using the generated grid method utilized for ligand docking calculations [36]. Ligand binding energy calculations with docked protein were calculated using the Prime MM-GBSA module panel [37].

### Cell viability assay

Cell viability of normal human fibroblasts (FF2441) and melanoma cell lines (UACC 903, 1205 Lu) following treatment with the active and inactive derivatives were measured by MTS assay (Promega, Madison, WI) [14, 27, 29–34]. In brief, 5 × 10^3^ cells per well in 100 μL of media were seeded and grown in a 96-well plate for 48 to 72 hours and treated with either DMSO vehicle control or 0.62 to 100 μmol/L of compounds for 72 hours. IC_50_ values for each compound in μmol/L for respective cell lines were measured from three independent experiments using GraphPad Prism version 4.01 (GraphPad Software, La Jolla, CA). In addition, recovery and viability assessment was also measured with these compounds co-treated simultaneously with 10 nmol/L of bafilomycin A1 (BafA1) or 1 mMol/L β-cyclodextrin and the viability was measured using the MTS assay after 24 hours [14].

### Assessing effects on endocytosis and lysosomotropism

The endocytotic capacity of the cells was measured as described in Kuzu et al. 2014 [14]. Briefly, UACC 903 cells were seeded into chamber slides and treated with the particular agents for 2 hours. Next, Alexa Flor 488 tagged transferrin protein was added at a final concentration of 5 μg/mL and incubated for 30 minutes. Cells were then fixed on the slide with 4% paraformaldehyde and analyzed by fluorescence microscopy as described previously [14]. Measurement of compounds effects on lysosomotropism was assessed through the appearance of cellular vacuolization of UACC 903 cells growing at 70 to 80% confluency on 100 mm culture dishes compared to controls. For some experimental conditions cells were co-treated with 10 nmol/L of BafA1 for 3 hours. Light microscopic images were used to assess cellular vacuolization [14].

### Western blot analysis

Cell lysates were harvested and processed as described in refs [14, 29, 31, 34]. In brief, 1.0 × 10^6^ melanoma cells were seeded in 100 mm culture dishes, 48 hours later; cells were treated with inactive derivatives of abietic acid, 2a & 5c (30–50 μmol/L) or active derivatives of leelamine, 4a & 5a (3–5 μmol/L) for 24 hours. Protein lysates were collected for Western blotting analysis. Blots were probed with antibodies according to each supplier's recommendations: antibodies to IGF-1R, total AKT, phosphor AKT (Ser473), total PRAS40, phospho-PRAS40 (Thr246), total ERK1/2, phospho-ERK1/2 (Thr202/Tyr 204), total CDK2, phospho-CDK2 (Thr160), p62, LC3B, total STAT, phospho-STAT3 (Tyr705), caspase 3 and cleaved PARP from Cell Signaling Technology (Danvers, MA); cyclin D1, α-enolase and secondary antibodies conjugated with horseradish peroxidase from Santa Cruz Biotechnology (Santa Cruz, CA). Immunoblots were developed using the enhanced chemiluminescence (ECL) detection system (Thermo Fisher Scientific, Rockford, IL).

### Dose escalation for oral administration of leelamine

To determine the effective oral dosage of leelamine, a dose escalation study was conducted in 4–6 week old Swiss Webster mice. Leelamine was dissolved in a tricaprylin microemulsion for oral administration and dosed at 40–160 mg/kg body weight every day for 14 days (3 mice/group). Animals were weighed daily to ascertain potential toxic effects leading to changes in body weight. At the end of treatment, blood was collected from each sacrificed animal in a serum separator tube with lithium heparin (BD Microtainer) following cardiac puncture and levels of serum biomarkers of major organ toxicity were measured. In addition, vital organs including liver, spleen, kidney, intestine, lung and heart from control and experimental animals were collected on day 14, formalin fixed, paraffin-embedded, H&E stained and analyzed microscopically for changes in cellular morphology or tissue architecture.

### Pharmacokinetics of leelamine in the serum of mice

Leelamine was extracted from serum as reported previously [12]. Swiss Webster mice were treated orally with 80 mg/kg body weight of leelamine, animals were euthanized and blood drawn by cardiac puncture at various time periods over 36 hours. Samples were kept at room temperature for 30 minutes followed by serum separation by centrifugation for 5 minutes at 5,000 rpm. Next, 20 μL of the collected serum was added to 80 μL of acetonitrile along with 5 μL of propranolol (RS)-1-(1-methylethylamino)-3-(1-naphthyloxy)propan-2-ol), which served as an internal standard (transition of m/z 259.9 to 116.0). Solution was vortexed for 30 seconds and then centrifuged at 10,000 rpm for 10 minutes. Supernatant was transferred to the auto sampler vials and subject to liquid chromatography-mass spectrometry using LC-MS/MS 2010 EV system (Shimadzu, Tokyo, Japan).

### Tumorigenicity assessment

1 × 10^6^ UACC 903 cells in 0.2 mL of DMEM supplemented with 10% FBS were subcutaneously injected above both left and right rib cages of 4–6 week old female athymic nu/nu mice. Six to eight days later, mice were randomly sorted into vehicle control 10% PEG (Poly ethylene glycol) and experimental compound 4a or leelamine groups, followed by daily oral administration of 80 mg/kg body weight. Volume of developing tumors in mm^3^ was measured on alternate days using calipers along with assessment of body weights [12, 13, 34].

### Sub-chronic toxicity assessment

At the end of tumorigenicity assessment, blood was collected from each euthanized animal in a serum separator tube with lithium heparin (BD Microtainer), following cardiac puncture and analyzed for levels of ALT (Alanine aminotransferase), AST (Aspartate aminotransferase), ALKP (Alkaline phosphatase), ALB (Albumin), GLB (Globulin), TPR (Total Protein), TBIL (Total bilirubin), BUN (Blood urea nitrogen), GLU (Glucose), CK (Creatine kinase), CAL (Calcium) [12, 13, 34].

### Statistical analysis

Statistical analysis was undertaken using the One-way/Two-way ANOVA GraphPad PRISM Version 4.01 software. Results were considered significant at a *p*-value of < 0.05.

## SUPPLEMENTARY MATERIALS FIGURES AND TABLE


