# Supplementary material for: Identifying the structure-activity relationship of leelamine necessary for inhibiting intracellular cholesterol transport
Source: Oncotarget. 2017 Mar 8;8(17):28260–77. doi: 10.18632/oncotarget.16002 (PMC5438648; doi:10.18632/oncotarget.16002)
Supplement: Supplementary file 1 [file oncotarget-08-28260-s001.pdf]

Identifying the structure-activity relationship of leelamine necessary for inhibiting intracellular cholesterol transport

Supplementary Materials

Leelamine

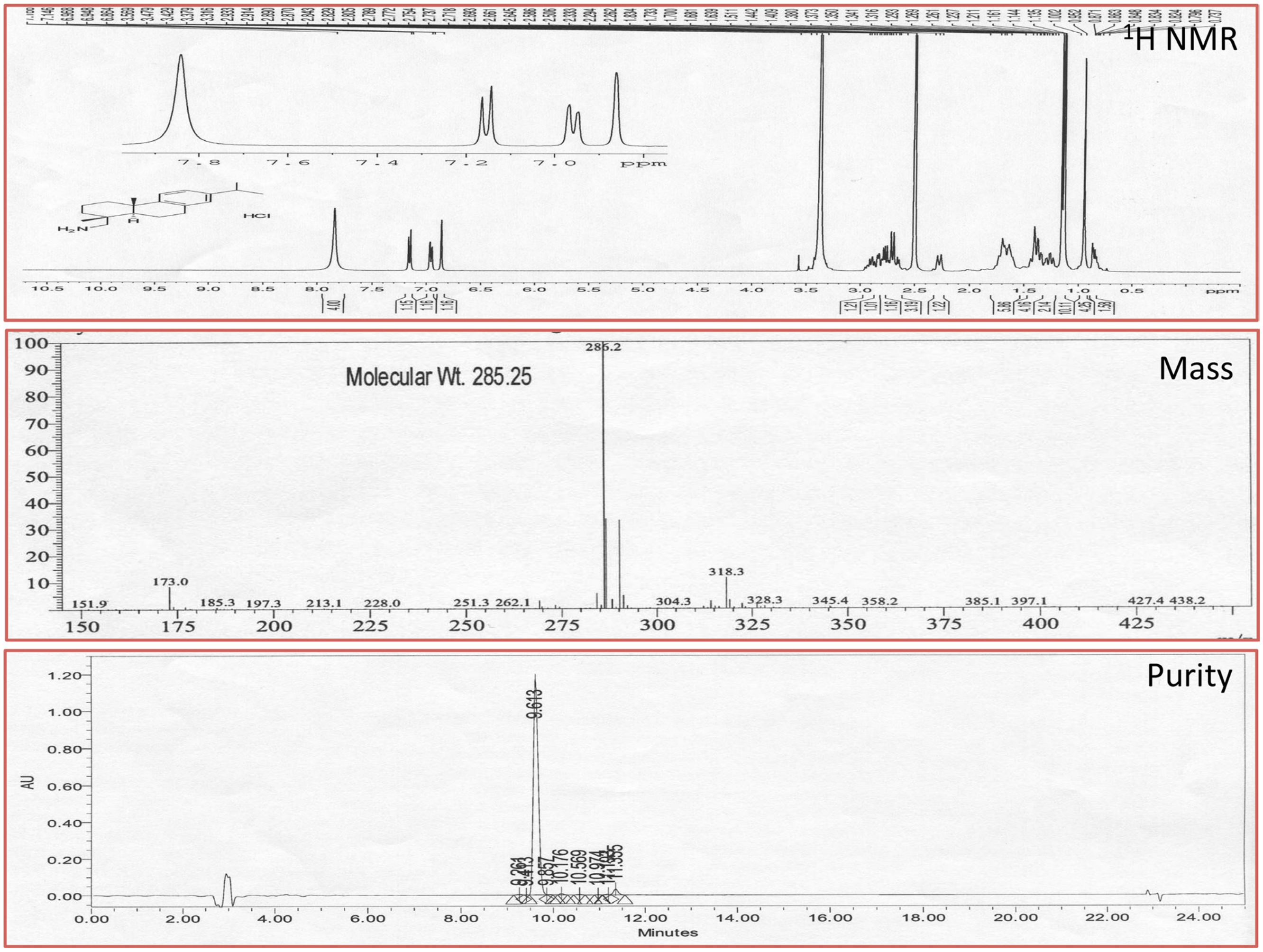

Compound 2a

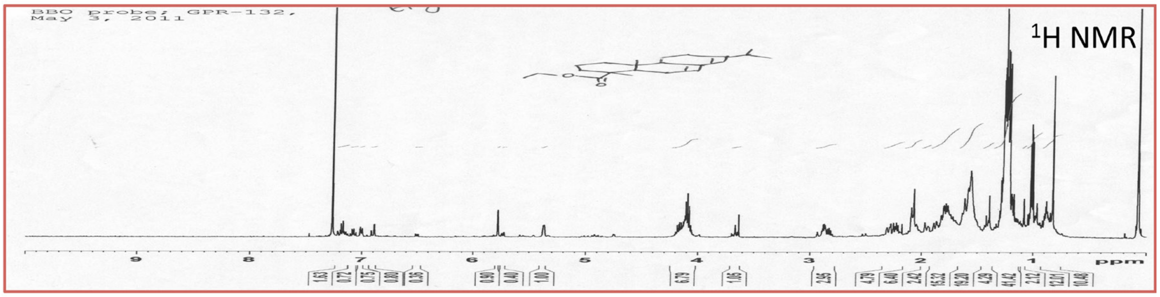

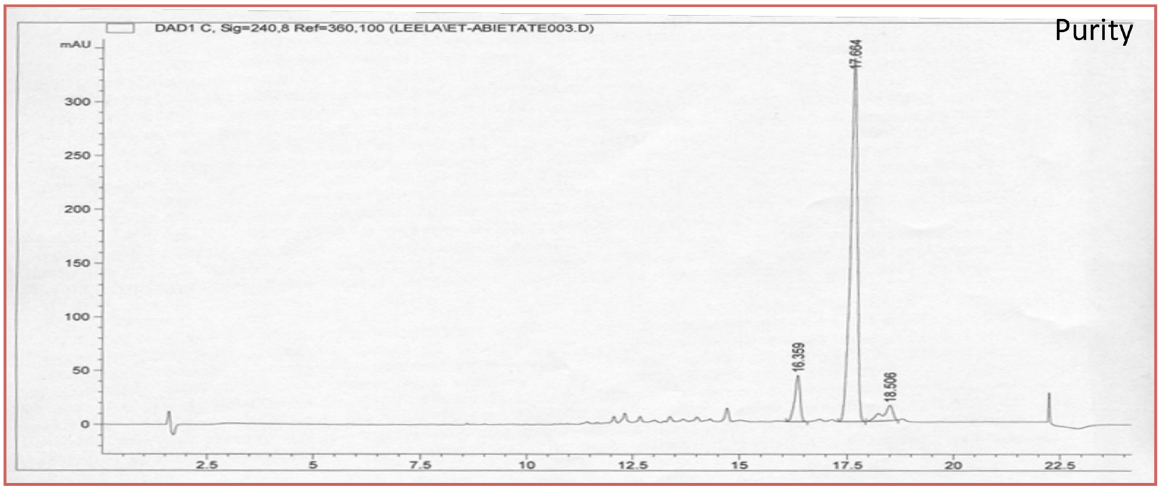

Compound 4a

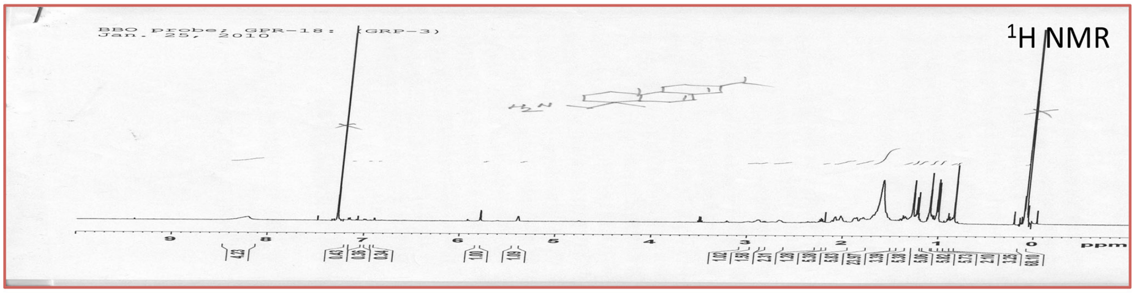

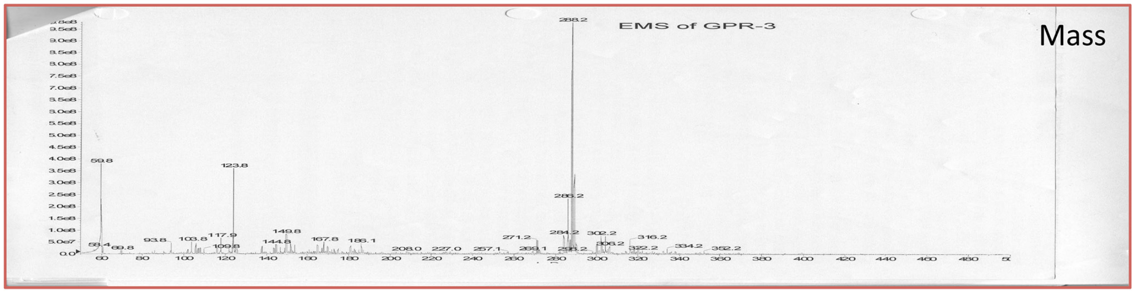

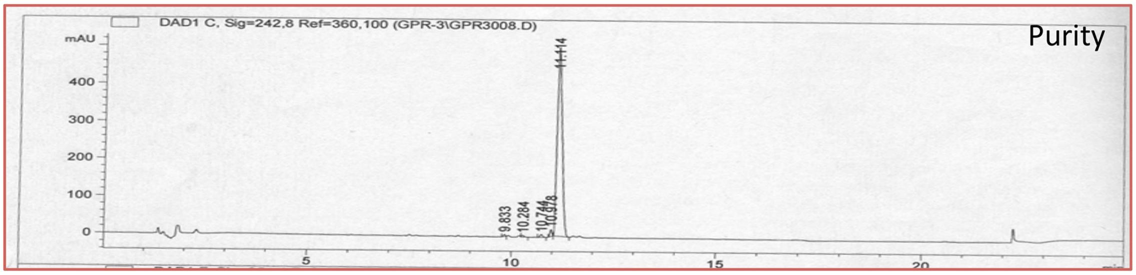

Compound 5a

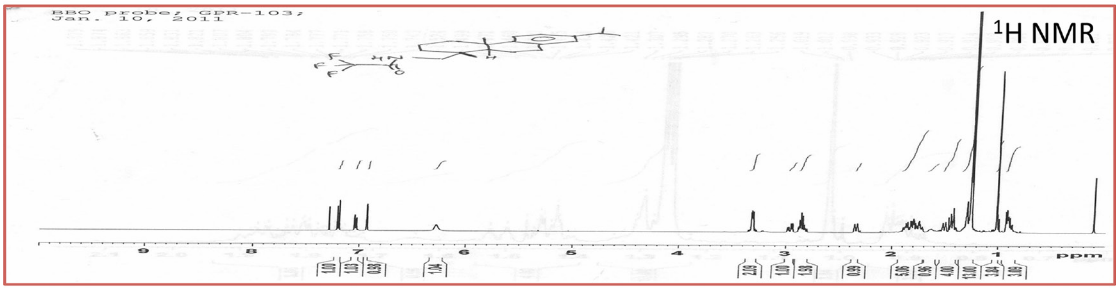

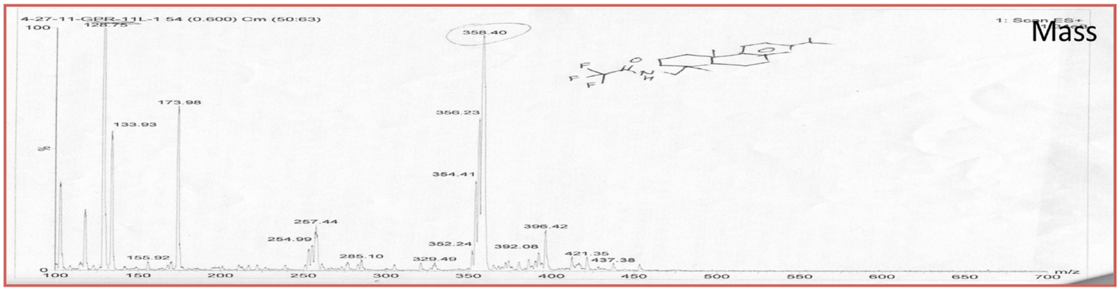

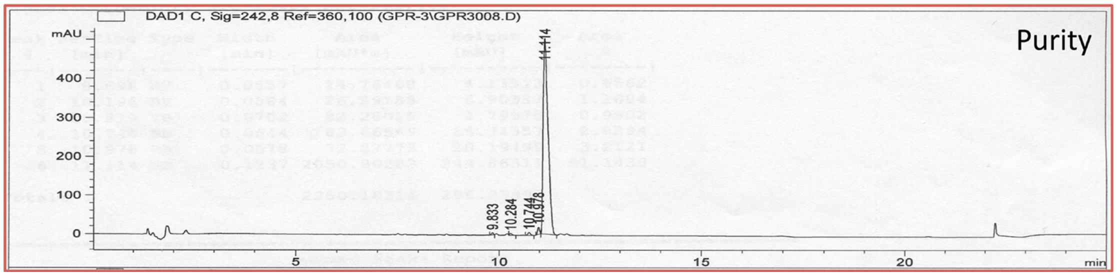

Compound 5c

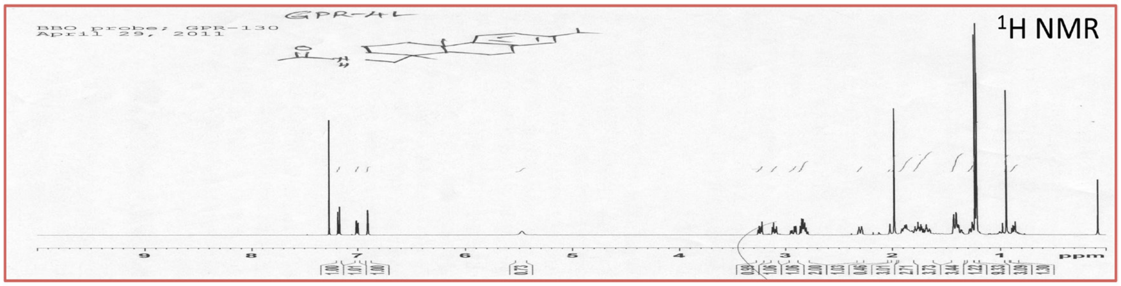

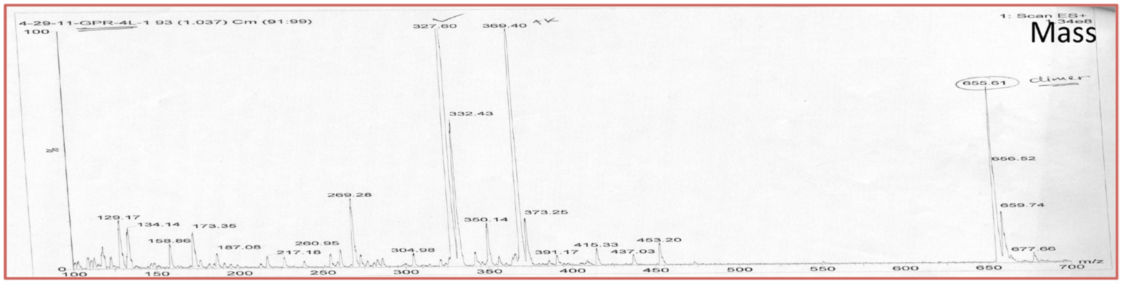

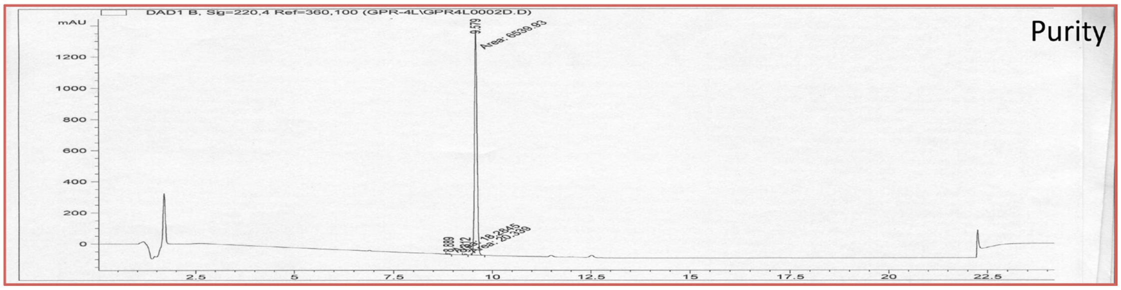

Supplementary Figure 1: Spectral and HPLC purity data for the key compounds leelamine, 2a, 5c, 4a and 5a.

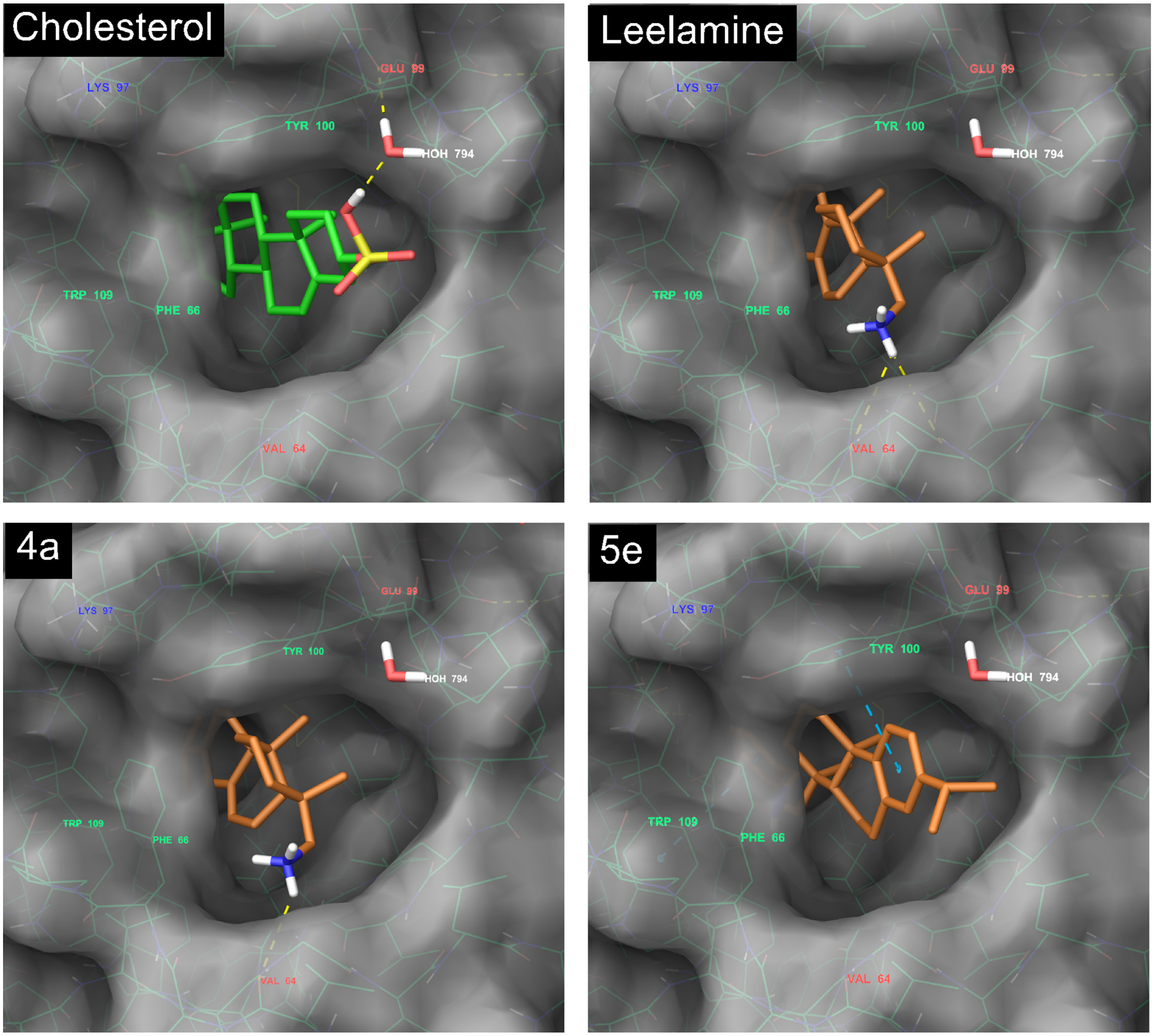

Supplementary Figure 2: Docking position of cholesterol, leelamine and compounds 4a and 5e in the ligand-binding pocket of NPC2 protein (PDB ID: 2HKA).

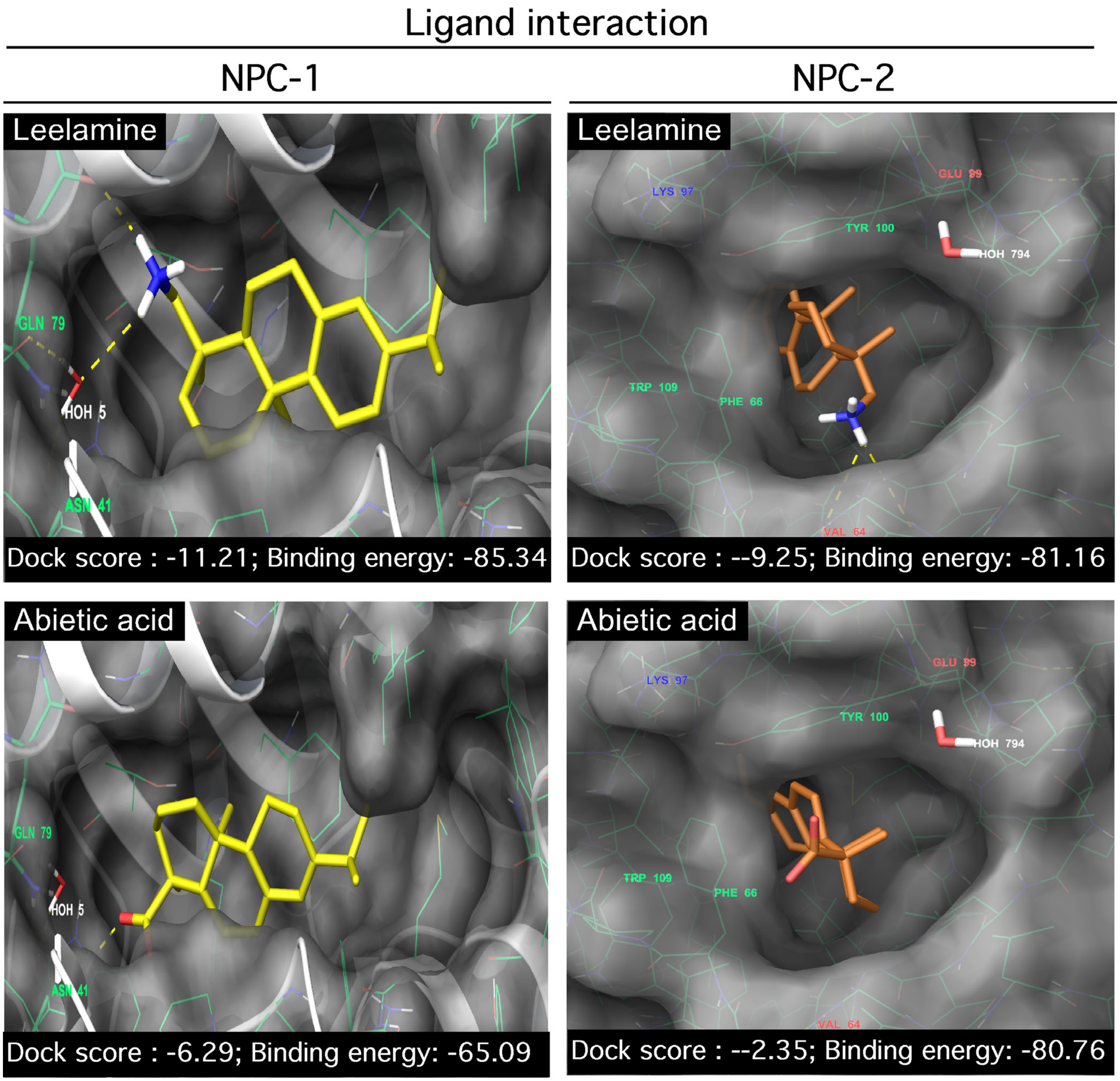

Supplementary Figure 3: Abietic acid weakly interacts with NPC1 and NPC2. Docking position of abietic acid in the ligand-binding pocket in NPC1 (PDB ID: 3GKI) or NPC2 (PDB ID: 2HKA). Leelamine served as the docking positive control.

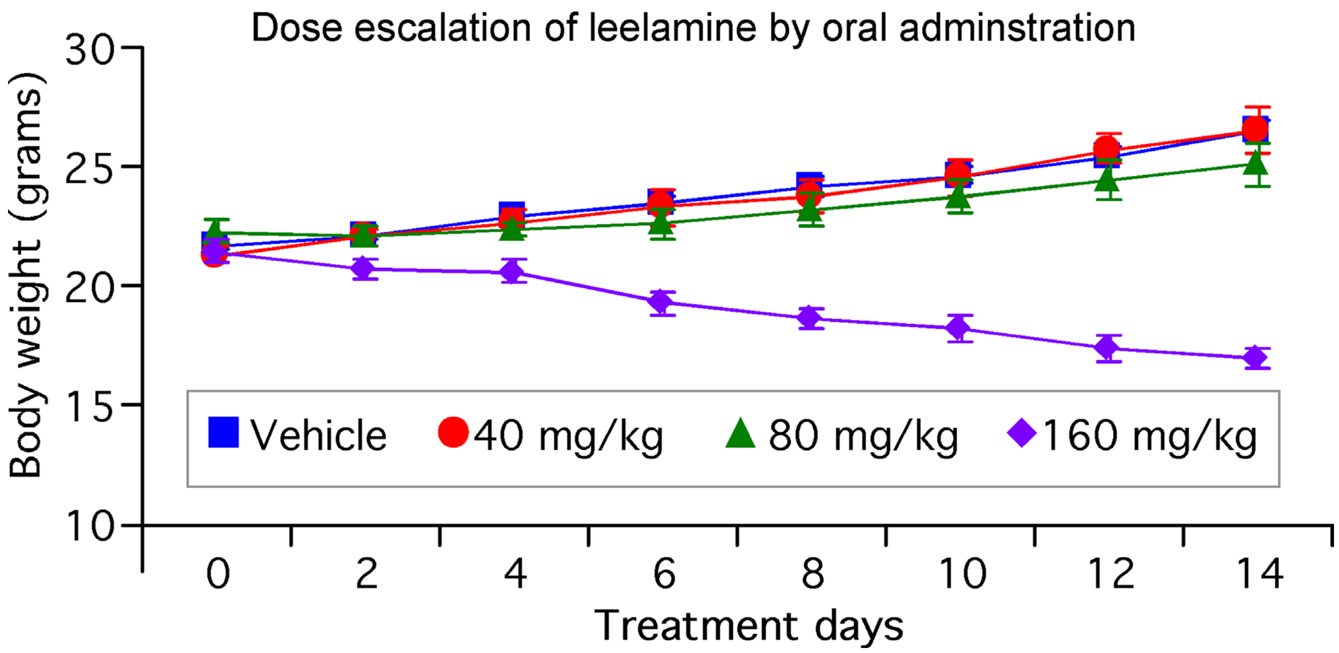

Supplementary Figure 4: Dose escalation for oral administration of leelamine in Swiss Webster mice. Leelamine was dissolved in a tricaprylin micro-emulsion for oral administration of 40-160 mg/kg body weight daily for 14 days (QOD). Animals were weighed on alternate days to ascertain changes in body weight suggestive of toxicity.

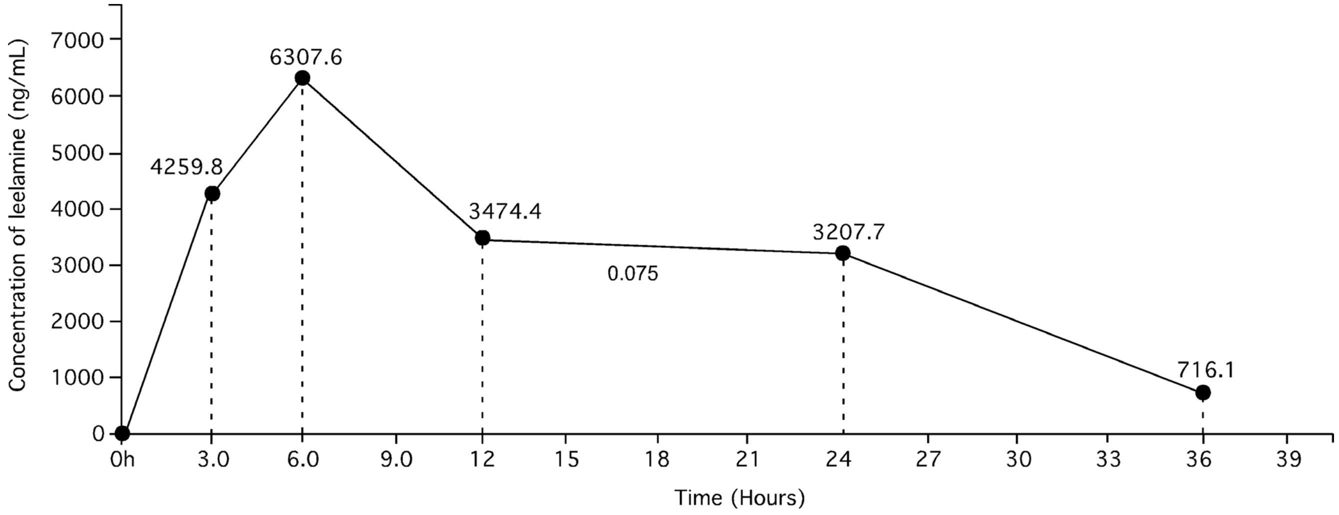

Supplementary Figure 5: Pharmacokinetics of leelamine in the serum of mice. Presence of leelamine in the serum of mice was measured following oral administration of 80 mg/kg body weight of leelamine over a period of 36 hours. Leelamine was present in the serum of mice for upto 24 hours and maximum concentration of leelamine was observed at 6 hours following oral administration.

```
CLUSTAL O(1.2.1) multiple sequence alignment

sp|P79345|NPC2_BOVIN      MRFLTVAFLFLALSASALAEPVKFKDCGSWVGVIKEVNVSPCPTQPCKLHRGQSYSVNVT
sp|P61916|NPC2_HUMAN      MRFLAATFLLLALSTAAQAEPVQFKDCGSVDGVIKEVNVSPCPTQPCQLSKGQSYSVNVT
                          ****:.:**:****::* ****:******  ****************:* :*********

sp|P79345|NPC2_BOVIN      FTSNTQSQSSKAVVHGIVMGIPVPFPIPESDGCKSGIRCPIEKDKTYNYVNKLPVKNEYP
sp|P61916|NPC2_HUMAN      FTSNIQSKSSKAVVHGILMGVPVPFPIPEPDGCKSGINCPIQKDKTYSYLNKLPVKSEYP
                          **** **:*********:**:******** *******.***:*****.*:******.***

sp|P79345|NPC2_BOVIN      SIKVVVEWELTDDKNQRFFCWQIPIEVEA--
sp|P61916|NPC2_HUMAN      SIKLVVEWQLQDDKNQSLFCWEIPVQIVSHL
                         ***:****:* ***** :***:**::: :
```

Supplementary Figure 6: Sequence alignment for homology modeling of bovine NPC2 protein (PDB ID: 2HKA).

Supplementary Table 1: Docking scores for active derivatives against NPC1 and NPC2

Docking scores and binding energies (KCal/mol)				
**Names**	**NPC1 (3GKI)**		**NPC2 (2HKA)**	
	**Docking score (XP GScore)**	**Bindning energy (MMGBSA)**	**Docking score (XP GScore)**	**Bindning energy (MMGBSA)**
**Cholesterol**	−14.35	−156.64	−11.01	−140.33
**Leelamine**	−11.21	−85.34	−9.25	−81.16
**4a**	−10.65	−101.12	−9.89	−95.87
**4b**	−10.78	−105.08	−9.30	−100.86
**5a**	−11.03	−83.71	−7.52	−92.98
**5b**	−10.66	−95.02	−10.02	−104.87
**5e**	−11.42	−78.59	−10.29	−91.69
